# Human milk extracellular vesicles target nodes in interconnected signalling pathways that enhance oral epithelial barrier function and dampen immune responses

**DOI:** 10.1002/jev2.12071

**Published:** 2021-03-10

**Authors:** Marijke I. Zonneveld, Martijn J.C. van Herwijnen, Marcela M. Fernandez‐Gutierrez, Alberta Giovanazzi, Anne Marit de Groot, Marije Kleinjan, Toni M.M. van Capel, Alice J.A.M. Sijts, Leonie S. Taams, Johan Garssen, Esther C. de Jong, Michiel Kleerebezem, Esther N.M. Nolte‐’t Hoen, Frank A. Redegeld, Marca H.M. Wauben

**Affiliations:** ^1^ Department of Biomolecular Health Sciences Faculty of Veterinary Medicine Utrecht University Utrecht The Netherlands; ^2^ Division of Pharmacology Department of Pharmaceutical Sciences Faculty of Science Utrecht University Utrecht The Netherlands; ^3^ Host‐Microbe Interactomics Group Department of Animal Sciences Wageningen University Wageningen The Netherlands; ^4^ Division of Infectious Diseases & Immunology Department of Biomolecular Health Sciences Faculty of Veterinary Medicine Utrecht University Utrecht The Netherlands; ^5^ Department of Experimental Immunology Academic Medical Center Amsterdam The Netherlands Centre for inflammation University of Amsterdam Amsterdam Infection & Immunity Institute (AI&II) Amsterdam The Netherlands; ^6^ Centre for Inflammation Biology and Cancer Immunology Department of Inflammation Biology School of Immunology & Microbial Sciences King's College London London UK; ^7^ Global Centre of Excellence Immunology Danone Nutricia Research Utrecht The Netherlands

**Keywords:** breast milk, exosomes, extracellular vesicles, gastrointestinal tract, human milk, immune modulation, immune system development, oral cavity, T cell modulation, TLR modulation

## Abstract

Maternal milk is nature's first functional food. It plays a crucial role in the development of the infant's gastrointestinal (GI) tract and the immune system. Extracellular vesicles (EVs) are a heterogeneous population of lipid bilayer enclosed vesicles released by cells for intercellular communication and are a component of milk. Recently, we discovered that human milk EVs contain a unique proteome compared to other milk components. Here, we show that physiological concentrations of milk EVs support epithelial barrier function by increasing cell migration via the p38 MAPK pathway. Additionally, milk EVs inhibit agonist‐induced activation of endosomal Toll like receptors TLR3 and TLR9. Furthermore, milk EVs directly inhibit activation of CD4+ T cells by temporarily suppressing T cell activation without inducing tolerance. We show that milk EV proteins target key hotspots of signalling networks that can modulate cellular processes in various cell types of the GI tract.

In the neonate, the epithelial barrier of the GI tract needs to grow and mature, while the adaptive immune system is still developing (Renz et al., 2011). This requires cellular regulation and education which, in part, is induced by components in mother's milk. Although the importance of breastfeeding is widely recognized, the milk components and molecular processes involved in these developmental processes remain largely obscure (Victora et al., 2016). This limited understanding is mainly due to the complexity of milk as it is composed of a wide range of bioactive macromolecular structures with partially overlapping physical properties (Ballard & Morrow, 2013; Chatterton et al., 2013; van Herwijnen et al., 2016). Among these components are extracellular vesicles (EVs) (Admyre et al., 2007; van Herwijnen et al., 2016; Zonneveld et al., 2014), which are nanosized particles released by cells. The release of EVs and the incorporation of molecular cargo into EVs is tightly regulated by the producing cell, resulting in a great heterogeneity of EVs specifically tailored for targeted intercellular communication (Mathieu et al., 2019; Robbins & Morelli, 2014). Altogether this contributes to the versatile capacity of EVs to modulate cellular responses at multiple levels. The multi‐faceted nature of EVs allows for the fine‐tuning of complex signalling pathways in order to control the magnitude, kinetics and duration of cellular responses (Raposo & Stahl, 2019; Steenbeek et al., 2018). Previously, we have established an isolation procedure specifically designed for the reliable isolation of EVs from human milk (Zonneveld et al., 2014) and performed in‐depth proteomics analysis of milk EVs (van Herwijnen et al., 2016). We discovered several milk EV‐associated proteins not identified before in human milk, that potentially contribute to both the development of the epithelial barrier and maintenance of immune homeostasis (van Herwijnen et al., 2016). In this study, we explored the physiological role of milk EVs in these processes and linked their protein cargo to underlying signalling pathways.

In order to isolate pure milk EVs we removed cells and cream from fresh (non‐frozen) breast milk before biobanking. We then used differential centrifugation followed by density gradient separation and Size Exclusion Chromatography (SEC) to obtain EVs in the desired culture medium for downstream analysis (Figure 1a). Importantly, the concentration of EVs in the final sample was comparable to that of EVs in milk, allowing us to study EVs in their physiological concentration. The milk matrix also contains several non‐EV components that can influence cells of the GI tract and might be co‐isolated.

**FIGURE 1:**
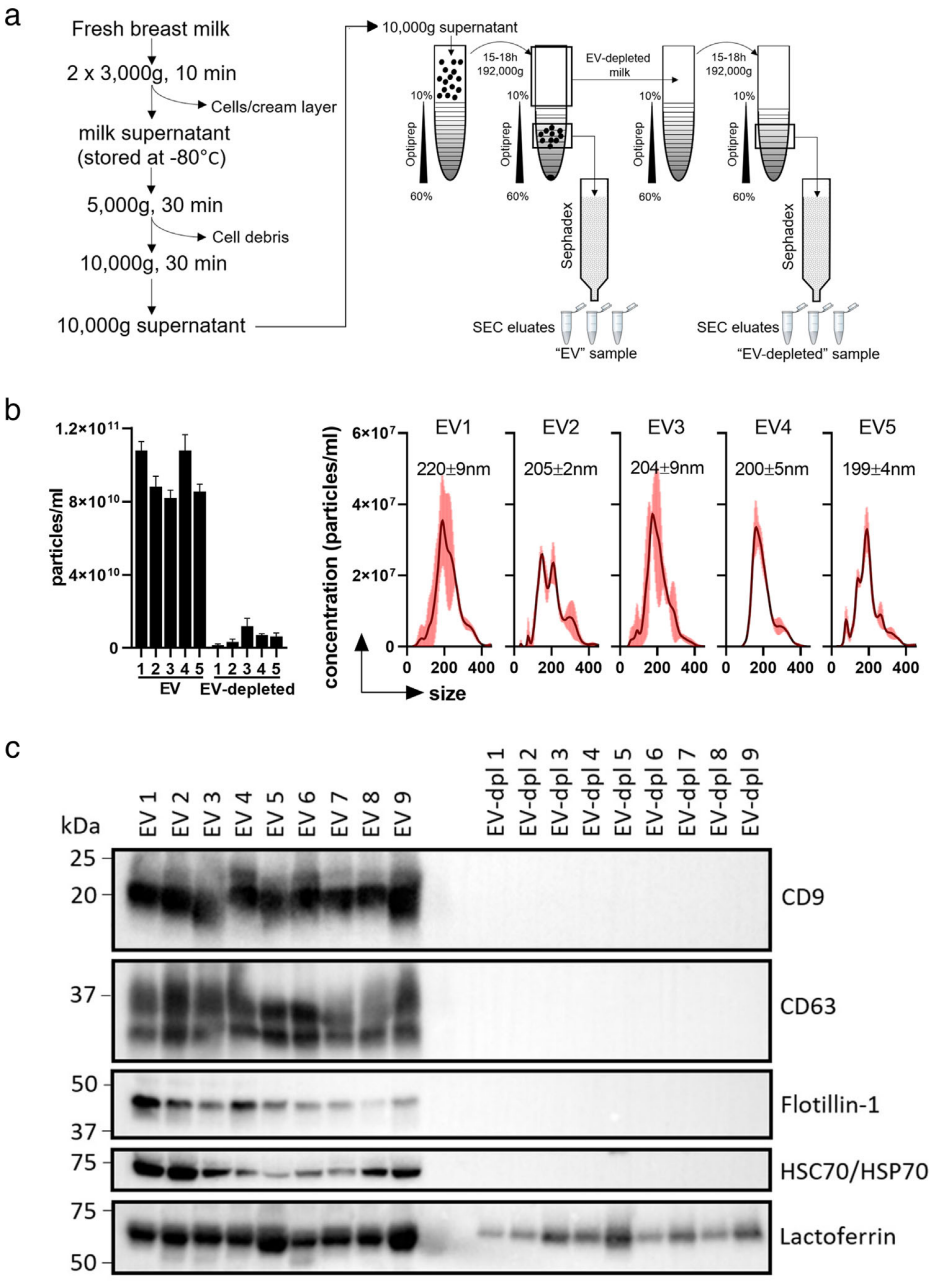
Isolation and characterization of human milk EVs and EV‐depleted milk control. [a) Schematic overview of the isolation of milk EVs and the EV‐depleted milk control using differential centrifugation followed by density gradient ultracentrifugation. The original isolation procedure (Zonneveld et al., 2014) applied sucrose as a density medium. However, to maintain functionality of isolated EVs, Optiprep was used to build the gradient for ultracentrifugation. Size exclusion chromatography (SEC) using Sephadex was applied to separate EVs from Optiprep and to collect the ‘EV fractions’ in the culture medium needed for functional analysis. The concentration of EVs used in the in vitro assays were within the physiological range, since the starting volume of 10,000 g milk supernatant was 6.5 ml prior to density gradient separation, while the final volume of the EV sample was 7 ml. The final volumes of EVs applied in the various assays varied between 75%–100% (see methods for more details). As a donor‐matched procedural control, we used the EV‐depleted milk supernatant from the first density gradient and applied this sample to a new density gradient after which fractions matching EV densities were collected and applied to SEC in order to obtain the final EV‐depleted control. b) Concentration measurements and particle size distribution of isolated milk EVs and their respective EV‐depleted controls from five donors. Flow mode measurements were performed with a NanoSight NS300 equipped with a NanoSight Syringe Pump and an optimized particles/frame rate in the range of 70–110 was used. Each measurement consisted of triplicate 60‐s captures. Mean and SD of three captures are shown. Particle distribution histograms of milk EV samples show size distributions in nm. c) The pooled SEC eluates that were used as EV or EV‐depleted samples were characterized by Western blot for the presence of EV‐associated markers CD9 (exposure time 1 s), CD63 (exposure time 5 s), Flotillin‐1 (exposure time 120 s), Hsp70 (exposure time 30 s) and non EV‐associated marker lactoferrin (exposure time 10 s) (data is from 1 experiment with a single sample from nine different milk donors)]

Additionally, our isolation procedure might introduce artefacts affecting cellular read‐out systems. Therefore, we also prepared a donor‐matched procedural control, called EV‐depleted, to validate any EV‐mediated effects (Figure 1a). To quantify the amount of EVs, we performed nanoparticle tracking analysis (NTA) and observed a substantial number of particles in the EV samples (average 9.3 × 10^10^ ± 1.2 × 10^10^ particles/ml; Figure 1b). In contrast, the EV‐depleted controls contained much less particles with fewer valid tracks and altered light scattering properties (Supplementary File 1 and Supplementary Figure 1). The size distribution of the isolated milk EVs ranged between 199–220 nm, albeit differences between the individual samples were observed, reflecting the heterogeneity of the milk EV population (Figure 1b). Next, we confirmed the presence of EV‐associated markers CD9, CD63, Flotillin‐1 and HSP70 in the milk EVs by Western blot analysis, while these markers were undetectable in the EV‐depleted controls (Figure 1c). In contrast, lactoferrin, an abundant soluble non‐EV milk protein was detected in both EV samples and their matched EV‐depleted controls, indicating the presence of milk matrix proteins in the procedural controls. Interestingly, although milk composition changes during lactation and can differ between mothers, we demonstrate rather limited inter‐donor variation in the EV characteristics. The might be due to the use of mature milk samples (average of 7.2 ± 3 months post‐partum) in which the variation in overall milk composition between mothers has becomes less (Ballard & Morrow, 2013).

Although often overlooked, the first interaction of milk components with the infant's mucosa occurs in the oral cavity, a site that needs to maintain an intact epithelial barrier and contains mucosa‐associated lymphoid tissue (MALT) (Groeger & Meyle, 2019; Moutsopoulos & Konkel, 2018). To determine whether milk EVs play a role in maintenance of the physical epithelial integrity, we performed a gap closure assay with gingival epithelial cells in the presence of milk EVs. Milk EVs significantly increased the re‐epithelialization rate of the epithelial cells to almost the same level as the positive control TGF‐α, while EV‐depleted milk supernatant did not (Figure 2a and 2b, Supplementary File 2). Re‐epithelialization can occur through p38 MAPK‐dependent migration (Huang, 2004; Sharma et al., 2003), either or not combined with cell proliferation through MEK‐ERK signalling (Mebratu & Tesfaigzi, 2009). To assess which milk EV proteins could be involved in the enhanced re‐epithelialization and which pathways were likely targeted, we performed enrichment, network‐ and functional annotation analysis on the milk EV proteome (van Herwijnen et al., 2016). Using enrichment analysis, we first identified 33 significantly enriched GO‐terms involved in cell cycle and migration to which collectively 159 proteins were associated (Supplementary File 3). Interestingly, network analysis revealed that 134 of these 159 proteins can form protein‐protein interactions (Supplementary Figure 2A), indicating that the combined EV cargo can be delivered as protein networks. Finally, functional annotation analysis was performed to link each protein from the identified protein networks to relevant signalling cascades involved in re‐epithelization, while also visualizing the expected mode of action of the milk EV protein (Supplementary Figure 2B). These analyses resulted in a model that shows that milk EV proteins can interact at multiple levels in signalling cascades and unveils several ‘hot spots’ where milk EV proteins formed nodes with a high number of potential interactions. Several of these nodes are formed around key cellular proteins that are involved in either inhibition of cell cycle (and proliferation), or stimulation of migration via p38 MAPK downstream of RAC1 and CDC42 (Supplementary Figure 2B and summarized in Figure 2c). To investigate whether milk EVs primarily affected re‐epithelialization by stimulating migration, cells were cultured in the presence of pharmacological inhibitors of p38 MAPK or MEK1/2 as a control for proliferation. Inhibition of p38 MAPK abolished the increased re‐epithelialization rate induced by EVs (Figure 2d), while MEK1/2 inhibition showed only a minor reduction of EV‐mediated re‐epithelialization (Figure 2e). To confirm the migratory behaviour of the epithelial cells, we performed single cell tracking to determine their displacement. In comparison to the cells cultured in medium or the EV‐depleted control, exposure to milk EVs caused a significant increase in cell displacement that was characterized by cells migrating longer tracks (Figure 2f and 2g). As re‐epithelialization is initiated instantly (Fernandez‐Gutierrez et al., 2017), this process is independent of substantial *de novo* gene transcription or protein production and is most likely due to regulation of the existing cellular proteome. We therefore investigated rapid changes in the expression of adhesion molecules, as our model predicted the involvement of E‐cadherin (hotspot in Supplementary Figure 2B) and EPCAM, which are involved in cell motility by remodelling the actin cytoskeleton (Barth et al., 2018; Shamir & Ewald, 2015). Indeed, we show that cell surface levels of E‐cadherin and EPCAM were significantly decreased in the presence of milk EVs (Figure 2h‐i and Supplementary Figure 3), which would allow for faster movement of the epithelial cells.

**FIGURE 2:**
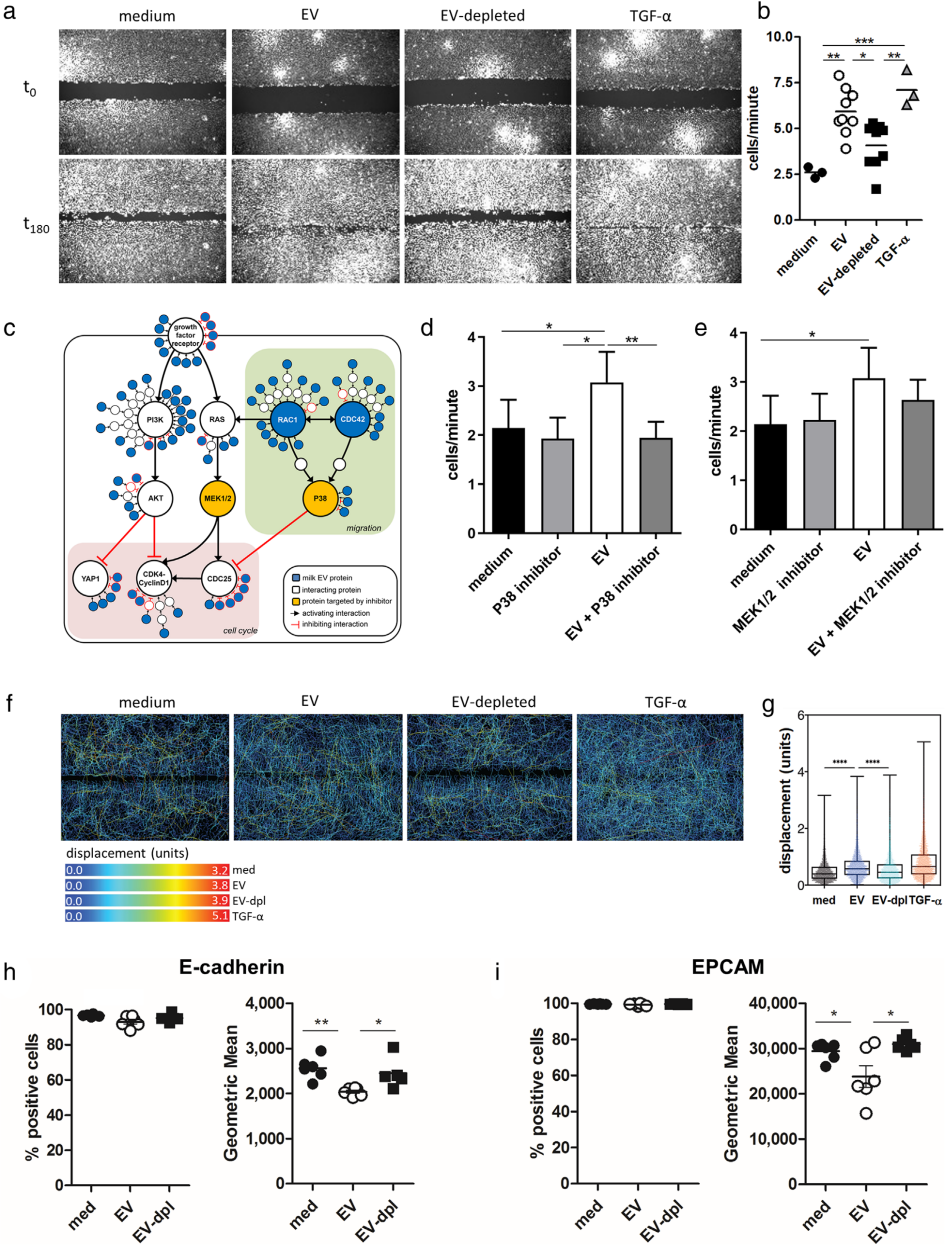
Human milk EVs enhance the formation of the epithelial barrier via p38 MAPK. [a) A gap was made in a confluent monolayer of Ca9‐22 gingival epithelial cells and re‐epithelialization was investigated in the presence of milk EVs, or milk donor‐matched EV‐depleted procedural control, or TGF‐α as a positive control. Cell migration was tracked in triplicate conditions by live cell imaging. Images were acquired every 20 min for 5 h, or until the gap in the TGF‐α condition was closed. Representative microscopic images of Ca9‐22 cells at the start of experiment (t0) and after 180 min (t180) incubation with indicated conditions are shown. Images are representative of two experiments with three milk donors per experiment, with triplicate technical replicates. b) Quantification of Ca9‐22 cells migrating into the gap, expressed in cells per minute during the linear growth phase. Each well was plotted as a single point and data are representative of two experiments with three milk donors in each experiment, with triplicate technical replicates. c) Schematic representation of key signalling pathways involved in gap closure. From the full annotation analysis (Supplementary file 3 and Supplementary Figure 2), key signalling pathways were selected that could be involved in gap closure. Several nodes with high interactions (≥6 interactions) were observed. These hotspots were involved in either i) inhibition of cell cycle (e.g., YAP1, CDK4‐CyclinD1 inhibition via the PI3K‐AKT pathway, or CDC25 inhibition via p38 MAPK pathway), ii) activation of cell cycle (e.g., MEK1/2 via RAS), or iii) stimulation of migration (e.g., the p38 MAPK pathway via RAC1 and CDC42). Proteins present in milk EVs are depicted in blue, while interacting cellular proteins are shown in white. Key proteins involved in cell cycle or migration that were inhibited in d and e are highlighted in yellow. Type of interactions between proteins is shown as activating or inhibiting. d and e) Quantification of Ca9‐22 cells migrating into the gap in the presence of p38 MAPK inhibitor (d) or MEK1/2 inhibitor (e). Bars represent mean ± SD. Data is representative of two independent experiments with two different milk donors per experiment, with triplicate technical replicates. f) From the experiment in figure a, the image series per treatment were converted into a hyperstack and single cells were tracked using TrackMate. Total track displacement is depicted by the pseudo‐coloured tracks. g) Summary of the cellular displacement per condition from figure f. h and i) Epithelial Ca9‐22 cells were cultured for 5 h in medium or together with milk EVs or EV‐depleted control after which cell surface expression of E‐cadherin (h) or EPCAM was determined (i). Shown are the percentage of E‐cadherin+ cells or EPCAM+ cells in the live gate and the Geometric mean of the cells positive for the respective marker. Data are representative of two experiments with three milk donors in each experiment, with a single technical replicate. Significance was calculated by Kruskal‐Wallis and Dunn's multiple comparison test, one‐way ANOVA and Tukey's multiple comparison test, or Brown‐Forsythe and Welch ANOVA using either Games‐Howell's correction for multiple comparisons with individual variances computed for each comparison or Dunnett's T3 post‐test and significance was defined as * *P* < 0.05; ** *P* < 0.01, *** *P* < 0.001, and **** *P* < 0.0001]

These data also fit previous observations that p38 MAPK regulates the post‐transcriptional expression of E‐cadherin (Lv et al., 2011; Zohn et al., 2006). Interestingly, others have shown a role for milk EVs of rat, porcine and bovine origin in modulating intestinal epithelial cells (Chen et al., 2016; Hock et al., 2017; Yu et al., 2017), suggesting that supporting epithelial integrity could be a central and evolutionary conserved function of maternal milk EVs. This idea is further supported by the presence of conserved milk EV miRNA cargo with regulatory functions that are shared between mammalian species, which could aid in this process (Herwijnen et al., 2018). Taken together, we demonstrate that human milk EVs directly enhance gingival epithelial cell migration via p38 MAPK and cytoskeletal remodelling. Milk EV proteins likely form clusters that can target migration‐related signalling cascades at multiple levels, thereby modulating the balance between key signalling nodes and tailoring cellular responses.

Epithelial cells lining the oral cavity also act as sentinels by scouting their environment via innate immune receptors. The activation of such receptors, for example, Toll‐like receptors (TLRs), needs to be critically regulated in order to enable the mucosal system to appropriately defend against pathogens, but tolerate commensals (Chung & Dale, 2008; Groeger & Meyle, 2019; Menckeberg et al., 2015). We therefore investigated whether milk EVs could be involved in TLR regulation.

Using TLR reporter cell lines, we observed that milk EVs and the EV‐depleted controls stimulated both TLR2 and TLR4 to some extent (Figure 3a and 3b), while TLR3 and TLR9 reporters were only triggered by their respective agonist (Figure 3c and 3d). Since milk is not sterile, and as milk collection and EV isolation might introduce microbial contaminations, we performed an endotoxin assay. We found that the milk EV samples, and to a lesser extent EV depleted controls, contained endotoxin (Supplementary Figure 4). Although it has previously been shown that EVs from other sources can stimulate TLR2 (Bretz et al., 2013), TLR3 (Seo et al., 2016), TLR4 (Bretz et al., 2013) and TLR9 (Ye et al., 2017), we show that milk EVs do not activate TLR3 and TLR9. Next, we determined whether milk EVs could regulate agonist‐induced TLR activation. Both TLR2 and TLR4 reporter lines showed equal stimulation in the presence of agonist regardless of the presence of milk EVs (Figure 3a and 3b). In contrast, milk EVs significantly dampened the agonist‐induced response of endosomal TLR3 and TLR9, while EV‐depleted controls had no or reduced effects (Figure 3c and 3d). To assess the inhibitory effect of milk EVs on endosomal TLR activation in a more physiological setting, we evaluated their effect on gingival epithelial cells. As these epithelial cells do not express TLR9, we assessed inhibition of TLR3 activation and found that also in these cells milk EVs reduced TLR3 activation, as measured by gene transcription of the pro‐inflammatory cytokines *IL6* and *CXCL8* (Figure 3e). The expression profile of other TLR‐related genes showed that in the presence of milk EVs, agonist‐induced gene upregulation could be fully inhibited (*LTA, REL, MAP2K4* and *IL6)*, or partially inhibited (*MAPK8, PELI1, FOS, JUN*, and *CXCL8*) (Figure 3f and Supplementary Figure 5 for complete gene array). Interestingly, the inhibition of *MAP2K4* (MEK4) and of its downstream targets *MAPK8* (JNK) and *JUN* supports the finding of epithelial cell migration via p38 MAPK (Figure 2), as MAP2K4 is predominantly involved in the activation of JNK and not p38 MAPK (Agarwal et al., 2015). In contrast to the inhibiting effects on gene expression, milk EVs enhanced the agonist‐induced upregulation of *EIF2AK2*, as well as that of *SIGIRR and SARM1*, both negative regulators of TLR signalling (Kondo et al., 2012; Liew et al., 2005). The enhancing effect of milk EVs was also observed for several genes that were downregulated by the agonist (*IRAK4, TLR6, PRKRA, PPARA* and *TLR3*). Finally, opposite effects of agonist‐induced activation were observed in the absence or presence of milk EVs (*TRAF6* and *TIRAP)* (Figure 3f). Collectively, the gene expression data indicate that milk EVs do not passively prevent TLR triggering by sequestering TLR ligands, but that they modulate TLR responses in an active manner. To assess which milk EV proteins could be involved in TLR modulation, we performed enrichment, network‐, and functional annotation analysis and identified 26 significantly enriched GO‐terms with 130 EV proteins associated to TLR signalling, of which 110 proteins formed protein‐protein networks (Supplementary File 3 and Supplementary Figure 6A).

**FIGURE 3:**
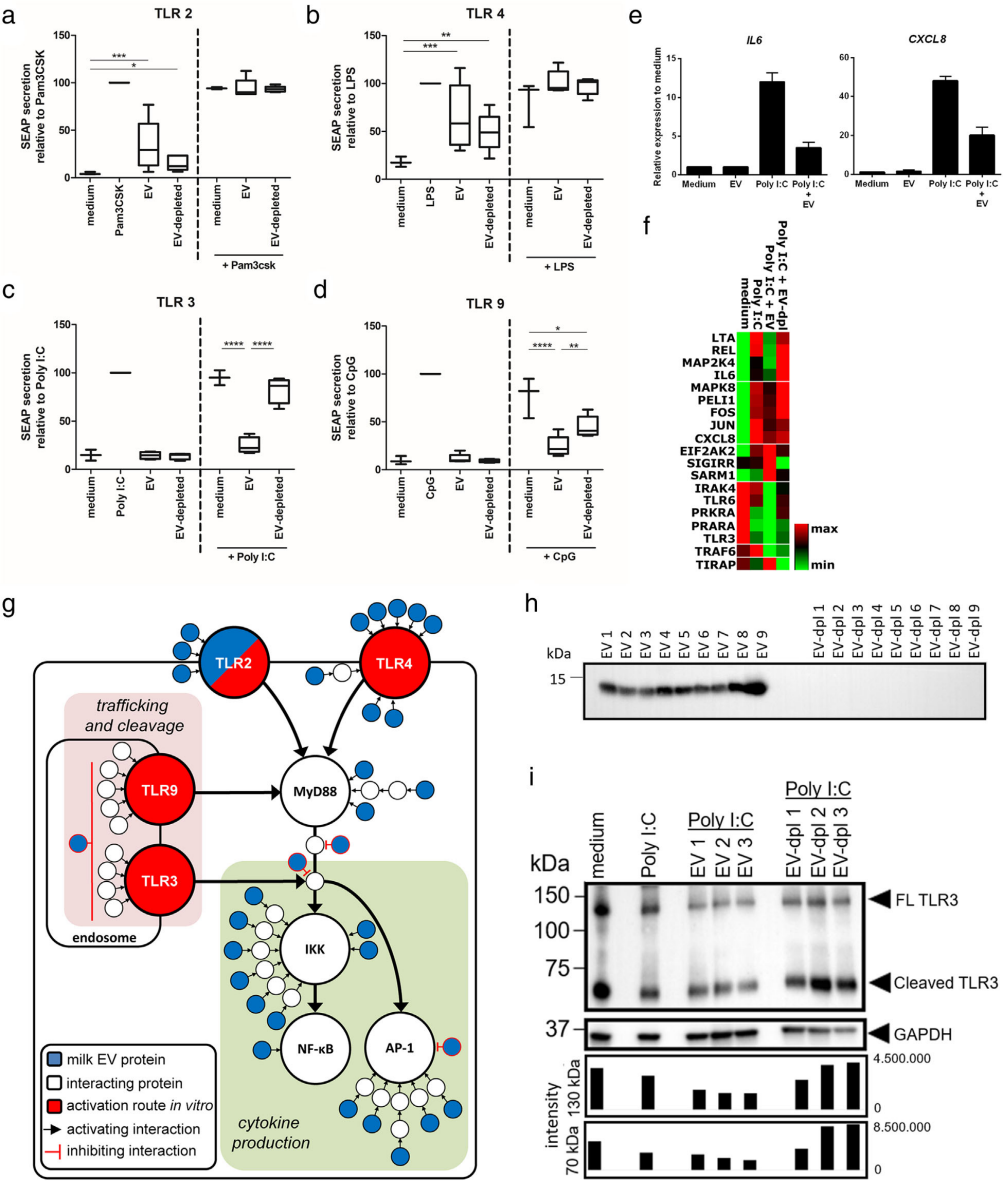
Human milk EVs inhibit agonist‐induced activation of endosomal TLR3 and TLR9, but not of cell surface TLR2 and TLR4. [a‐d) Secretion of SEAP reporter protein was determined for TLR reporter cell lines cultured in indicated conditions either or not in response to agonist (Pam3CSK for TLR2, LPS for TLR4, Poly I:C for TLR3, and CpG ODN2006 for TRL9), with the TLR‐specific agonist set to 100%. Box and whisker plots contain data from a single technical replicate of three independent experiments with a total of four different milk donors. e) Relative gene expression of *IL6* and *CXCL8* in Ca9‐22 cells cultured for 5 h with medium, EV, Poly I:C or Poly I:C + EV. Delta Ct‐values to *ACTB* and *GAPDH* were calculated and expressed relative to medium controls. EVs from two different milk donors were used and PCR reaction was performed twice on a single technical replicate. Results are summarized in bar graphs as mean ± SD. f) Heatmap representing the cellular gene expression profile of Ca9‐22 cells cultured for 4 h in the four different conditions (medium, agonist, EV + agonist, EV‐depleted + agonist) in a gradient running from minimal gene expression (green) to maximal expression (red) for each gene analyzed (see Supplementary Figure 5 for complete dataset). Data is from 1 technical replicate derived from 1 experiment, with 1 milk donor. g) Schematic representation of key signaling pathways involved in TLR signaling of the individual TLRs tested which are shown in one Figure. TLR2, TLR4 and TLR9 signal via MyD88, while TLR3 signals via TRIF (not shown) to NF‐kB (via IKK) and/or AP‐1 leading to cytokine production. TLR2 and TLR4 are surface receptors, while TLR3 and TLR9 are sorted into the endosome where they are cleaved which enhances their signaling. Proteins from milk EVs are depicted in blue, while interacting cellular proteins are shown in white. Type of interactions between proteins is either shown as activating or inhibiting. In every assay, the individual TLR was activated via its specific ligand, which is shown in red. Note that TLR2 is present in milk EVs as well as in the TLR2 reporter cells. h) Western blot analysis for the presence of Cystatin‐B (CSTB; expected size 11 kDa; exposure time 19 s) in purified milk EVs or in EV‐depleted control. Data is from one experiment with a single sample from nine different milk donors. i) Western blot analysis for the presence of TLR3 on whole cell lysate of Ca9‐22 cells cultured in medium alone or stimulated with poly I:C with or without milk EVs or EV‐depleted control. Full length (FL) TLR3 (expected size 130 kDa) and cleaved TLR3 (expected size 70 kDa) are visible. GAPDH (expected size 36 kDa) was used as a loading control and applied to normalize the intensity of the FL band and cleaved band (shown as volume intensity below the blot) in order to quantitatively compare signals. A total of three different milk donors were tested in a single experiment. Significance was calculated with 2‐way mixed model analysis and Bonferroni correction and significance was defined as * *P* < 0.05; ** *P* < 0.01; *** *P* < 0.001 and **** *P* < 0.0001]

From this analysis we extracted the key TLR signalling pathways targeted by milk EVs, leading to cytokine production or regulation of TLR trafficking and processing (Supplementary Figure 6B and Figure 3g). Besides the presence of low levels of endotoxin in the milk samples (Supplementary Figure 4), based on this model, the TLR2 and TLR4 activating effect of milk EVs in the absence of TLR agonists (Figure 3a and 3b) can be explained by the presence of several TLR2 and TLR4‐stimulating proteins associated to milk EVs. As the signalling cascades for both plasma membrane and endosomal TLRs converge, no distinctive regulatory mechanism could be identified in our model that explains the inhibition of endosomal TLRs signalling. However, in contrast to TLR2 and TLR4, endosomal TLRs require specific trafficking and processing which involves transportation into the endosome followed by proteolysis via cathepsins, which enhances their signalling (Ewald et al., 2008; Garcia‐Cattaneo et al., 2012; Gay et al., 2014). From our functional annotation analysis, we identified the cathepsin inhibitor cystatin‐B (CSTB) (Turk et al., 1997), and verified its presence in milk EVs using immunoblotting (Figure 3h). Although CSTB has not been reported to be involved in the modulation of endosomal TLR cleavage, we explored whether milk EVs could affect the unprocessed full length and the cleaved form of TLR3 after agonist‐induced activation of epithelial cells. Indeed, we observed less cleaved TLR3, however the abundance of full length TLR3 was also lower (Figure 3i). This suggests that milk EVs can modulate expression, or might interfere with TLR3 synthesis, for which the inhibition of *TLR3* gene expression is indicative (Figure 3f). Taken together, our data show that milk EVs selectively inhibit agonist‐induced endosomal TLR signalling, regulate downstream gene expression and reduce cellular full length and cleaved TLR3 expression. The selective regulation of key hotspots in TLR signalling could support the colonization of the mucosa in the new‐born, as it has been shown that differential NF‐κB activation is essential for immune homeostasis and tolerance to commensal bacteria (Chung & Dale, 2008; Lee et al., 2006).

In contrast to innate immune cells, cells from the adaptive immune system are educated during postnatal development, whereby CD4+ T cells located in the MALT differentiate into T helper (Th) and regulatory subsets (Masopust & Schenkel, 2013; Van Den Broek et al., 2018). Since epithelial cells can endocytose milk EVs (Liao et al., 2017) and selectively transport macromolecular structures from the apical to basolateral compartments via transcytosis (Tuma & Hubbard, 2003), it is assumed that milk EVs can reach cells underlying the epithelial barrier. Therefore, we evaluated the effect of milk EVs on T cell activation and differentiation. When CD4+ T cells were activated in the presence of milk EVs, they were halted in their proliferation (Figure 4a and 4b). This inhibitory effect was dose‐dependent (Figure 4c and Supplementary Figure 7) and transferrable to the EV‐depleted control (Figure 4d and Supplementary Figure 8). In order to determine whether milk EVs only interfered in proliferation and not differentiation, we monitored the ratio of naïve CD45RA+ to memory CD45RO+ T cells (Sallusto et al., 2004; Van Den Broek et al., 2018). As expected, activation of T cells skewed the T cell population towards CD45RO at the expense of CD45RA (Figure 4e and 4f). In contrast, addition of milk EVs during T cell activation prevented this switch and T cells retained CD45RA expression (Figure 4e and 4f).This effect was dose‐dependent (Supplementary Figure 9) and milk EVs were able to transfer this effect to the EV‐depleted control in a dose‐dependent manner (Supplementary Figure 10).The inhibitory effect of milk EVs was not merely via blocking proliferation of memory T cells (CD45RO), as proliferation of highly purified naïve CD4+CD45RA+ T cells was also inhibited (Supplementary Figure 11). This indicates that milk EVs inhibit activation of naïve T cells and their transition to a memory phenotype. Further evidence for impaired T cell activation in response to milk EVs was found by the inhibited release of a wide range of different Th subset‐associated cytokines, for example, IFN‐γ (Th1), IL‐5 (Th2), IL‐9 (Th2, Th9 and Th17), IL‐10 (Th2 and regulatory T cell: Treg), IL‐13 (Th2), IL‐17A and IL17F (Th17), and IL‐22 (Th1, Th17) (Figure 4g). Suppression of proliferation and cytokine production could be indicative for Treg induction (Van Den Broek et al., 2018), and it has been previously reported that a crude preparation of human milk EVs induced expression of the Treg‐associated transcription factor FoxP3 (Admyre et al., 2007). Yet, after exposure of activated purified T cells with a near physiological amount of milk EVs, we did not observe the induction of the typical Treg phenotype with high expression of CD25 and FoxP3 in the absence of CD127 (Supplementary Figure 12).

**FIGURE 4:**
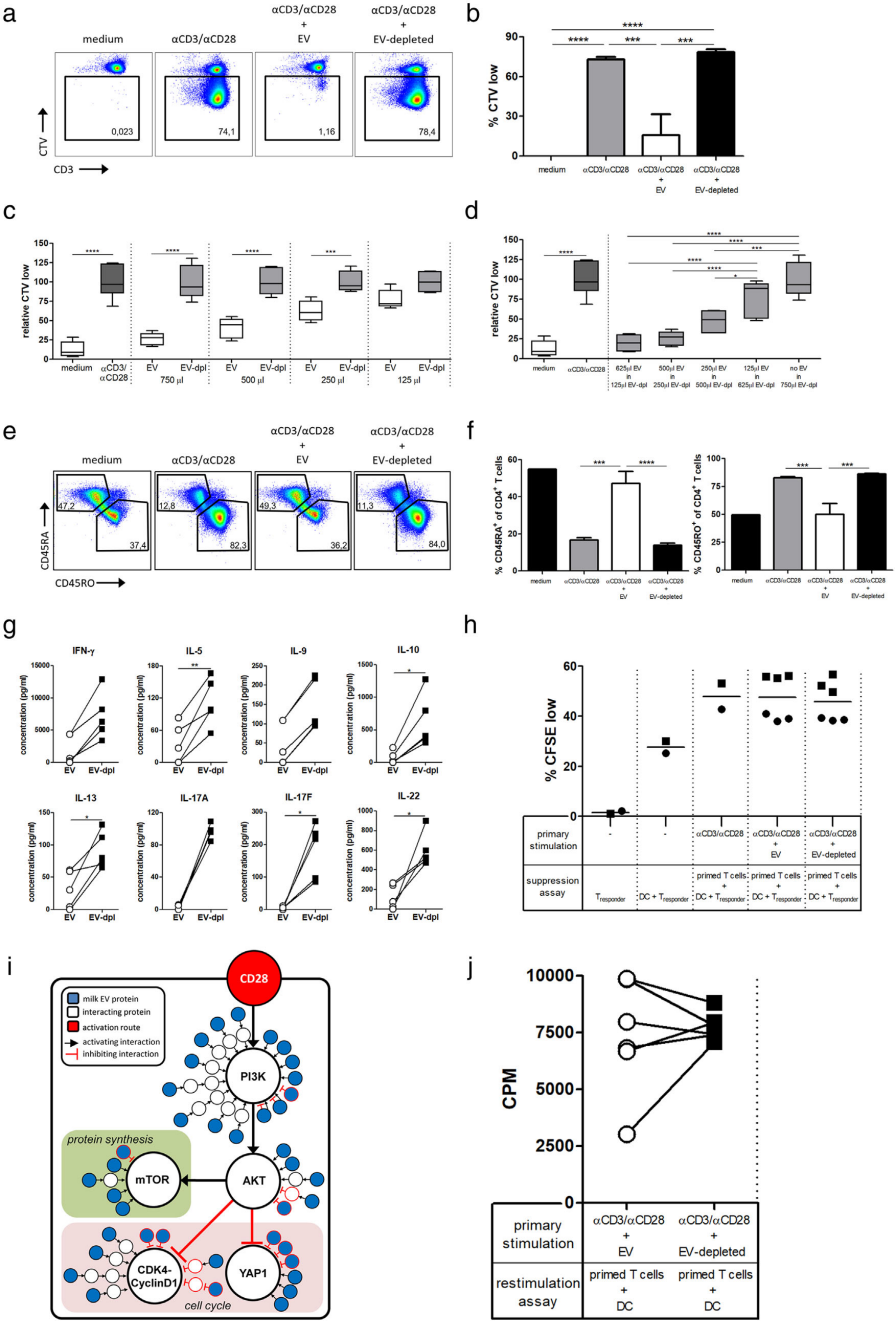
Human milk EVs transiently inhibit CD4+ T cell activation and retain cells in a naïve phenotype. [a‐f) Purified CD4+ T cells were labelled with Cell Trace Violet (CTV) and incubated with medium, αCD3 and αCD28 (αCD3/αCD28), or αCD3/αCD28 in the presence of EV or EV‐depleted control for 6 days. a) Representative dot plots of CTV dilution in response to the indicated conditions. Percentage CTV^low^ cells (gate) are expressed as a fraction of total CD4+ T cells. b) Quantification of the percentage CTV^low^ CD4+ T cells following incubation with the indicated conditions. Bars summarize mean ± SD of a single technical replicate of a representative experiment of two independent experiments using different T cell donors (n = 2) and different milk donors (n = 2 and 3). c and d) Relative expression of CTVlow CD4+ T cells following incubation with the indicated conditions, with each paired control set to 100%. Box and whisker plots contain data of a single experiment using n = 1 T cell donor and n = 3 different milk donors. e) Representative dot plots of CD4+ T cells stained for CD45RA and CD45RO after culture in the indicated conditions. Percentage of CD45RA+/CD45RO‐ and CD45RO+/CD45RA‐ cells in gates are expressed as a fraction of total CD4+ T cells. f) Quantification of the percentage CD45RA+ and CD45RO+ cells of total CD4+T cells following incubation with the indicated conditions. Bars summarize mean ± SD of a single technical replicate of a representative experiment of two independent experiments using different T cell donors (n = 2) and different milk donors (n = 2 and 3). g) Cytokine profiles of stimulated CD4+ T cells incubated with EV or EV‐deleted controls. The following values were obtained for αCD3/αCD28 stimulated controls (not shown in the bar graph), provided as mean ± SD: IFN‐γ: 4514 ± 1757 pg/ml, IL‐5: 89 ± 30 pg/ml, IL‐9: 123 ± 37 pg/ml, IL‐10: 330 ± 243 pg/ml, IL‐13: 99 ± 22 pg/ml, IL‐17A: 13 ± 8 pg/ml (compared to 97 ± 8 pg/ml with EV‐depleted), IL‐17F: 68 ± 47 pg/ml (compared to 180 ± 76 pg/ml with EV‐depleted), IL‐22: 346 ± 59 pg/ml. Data is shown for day 6 as highest secretion was seen for αCD3/αCD28 controls on this day. Graphs summarize results of a single technical replicate from two independent different T cell donors (n = 2) and different milk donors (n = 2 and 3). h) Suppression assay performed with CFSE‐labelled CD4+ T_responder_ cells incubated with allogeneic monocyte‐derived DC and autologous CD4+ T cells that received primary stimulation with αCD3/αCD28 or with αCD3/αCD28 in the presence of EVs or the EV‐depleted control. After priming, the cells were washed, irradiated and added to an allogeneic mixed lymphocyte reaction (MLR). Plotted are single points for the average of technical triplicates, shown as a percentage of total T_responder_ cells. Data is representative of a single technical replicate from two independent experiments (with two individual donors) using three different milk donors. i) Schematic representation of key signaling pathways involved in regulating T cell activation: downstream signaling of CD28 with inhibition of in cell cycle (e.g., YAP1 and CDK4‐CyclinD1 via PI3K‐AKT) and activation of mTOR (via PI3K‐AKT). Proteins from milk EVs are depicted in blue, while interacting cellular proteins are shown in white. Type of interactions between proteins is either shown as activating or inhibiting. CD28 is highlighted in red, as this receptor was stimulated. j) Restimulation assay with purified CD45RA+ CD4+ T cells (n = 2) that received a primary stimulation with αCD3/αCD28 in the presence of EVs or EV‐depleted control (n = 3). Cells were subsequently restimulated with allogeneic monocyte‐derived DC in the absence of EV and EV‐depleted controls. Restimulation was performed in triplicate and the average values plotted as a single point. Proliferation was measured by ^3^H‐thymidine incorporation and data is expressed as counts per minute (CPM). Significance was calculated with one‐way ANOVA and Tukey's or Sidak's multiple comparisons test (b, c and d; for f medium controls were not normally distributed and were therefore omitted from statistical analysis), paired *t*‐test, or Wilcoxon matched pairs signed rank test (g and j), or Kruskal‐Wallis and Dunn's multiple comparison test (h) and significance was defined as * *P* < 0.05; ** *P* < 0.01; *** *P* < 0.001 and **** *P* < 0.0001]

Additionally, we functionally tested the inhibitory capacity of T cells exposed to milk EVs. For this, CD4+ T cells were first stimulated in the presence of milk EVs, recovered and cultured with fresh CD4+ T‐responder cells. As expected, in this suppression assay the milk EV‐primed T cells were not suppressive (Figure 4h). To assess which pathways might be targeted by milk‐EV proteins in the observed inhibition of T cell activation, we used enrichment analysis and identified a total of 48 significantly enriched GO‐terms containing 165 proteins, of which 137 proteins were part of protein‐protein interaction networks (Supplementary File 3 and Supplementary Figure 13A). Using functional annotation analysis, we could link the majority of these interacting proteins to relevant signalling cascades downstream of CD28, resulting in the inhibition of cell cycle and the stimulation of mTOR (Supplementary Figure 13B and Figure 4i). Although we do not provide direct molecular evidence, this model provides an explanation for the inhibition of proliferation and cytokine production by milk EVs, as the inhibition of downstream CD28 signalling can result in the retained naïve CD4+ T cell phenotype (Harding et al., 1992). Furthermore, the absence of Treg induction by milk EVs becomes evident, as mTOR and PI3K inhibit FoxP3 expression in T cells (Merkenschlager & Von Boehmer, 2010). These findings suggest that milk EVs directly inhibit CD4+ T cell activation, without T cell tolerance or Treg induction. To test this, we activated naïve CD4+ T cells in the presence of milk EVs, recovered the cells and restimulated them with allogeneic DC. Indeed, T cells that were primed in the presence of milk EVs, proliferated after restimulation, to the same extent as restimulated T cells primed with EV‐depleted control (Figure 4j). This demonstrates that the presence of milk EVs causes a transient inhibition of T cell activation which is reversible. Based on our findings we propose that milk EVs create a temporary increased threshold for CD4+ T cell activation. Raising this threshold might help the infant to cope with the high antigenic load to which it is exposed after birth, creating tolerogenic conditions that are optimal for development (Basha et al., 2014; Caballero & Pamer, 2015).

Although human milk is known to aid the postnatal development and maturation of the GI tract, and maintenance of immune homeostasis (Cummins, 2002; He et al., 2016; Renz et al., 2011; Turfkruyer & Verhasselt, 2015), the exact effects on the oral mucosa has remained understudied. Here we demonstrate by using *in vitro* model systems that purified EVs from human milk can promote gingival cell re‐epithelialization, modulate epithelial endosomal TLR responses and transiently control T cell activation (Supplementary Figure 14). As EVs are heterogeneous in composition, the net‐effect on the target cell will be determined by the collective EV cargo delivered and relevant pathways targeted. Therefore, EV‐induced effects result from the concerted action of numerous biomolecules delivered, rather than from a single regulatory molecule. This is underscored by our models derived from the functional annotation analysis of the milk EV proteome, in which potential interactions of protein cargo occurs throughout the signalling cascades and where nodes of high interaction affect key signalling proteins. Whereas some ‘regulatory hotspots’ like PI3K, are prominent in all three biological processes studied, other unique hotspots were identified in each biological assay. In the end, these models do not establish a causal link and therefore molecular or functional validation remains essential to understand how milk EV cargo targets cellular components. Besides proteins, milk EV contain other cargo like miRNAs, from which several miRNAs (let‐7 family members let‐7a, let‐7b, let‐7f, and miR‐148a) have validated target genes that are involved in signal transduction and cell growth and/or maintenance (Herwijnen et al., 2018). Therefore, milk EVs have the potential to not only regulate cellular processes on a protein level via their protein cargo, but can also do this on a transcriptional level for instance via their miRNAs. In that respect we postulate that for the biological function of EVs, heterogeneity of the EV population and their respective cargo ensures fine‐tuning with respect to specific cellular response. Future studies are needed to validate our *in vitro* findings *in vivo* and to determine which EV subsets interact with specific recipient cells, as it is likely that EV subsets will contain specific cargos that are geared towards distinct functions in target cells, and that their composition is controlled by environmental cues from the mother (Torregrosa Paredes et al., 2014).

In summary, EVs are multifactorial and bioactive components of human milk that can interact with various cell types found in the oral mucosa, creating a window of opportunity for regulated development of the epithelial barrier and the innate and adaptive immune system of the new‐born.

## 1 METHODS

### 1.1 Human milk collection

Human milk was collected as previously described (Zonneveld et al., 2014). Briefly, fresh and mature milk samples were collected by means of an electric breast pump by 16 healthy mothers, with a mean age of 33 ± 2.4 years who were at a lactational stage of 3 to 15 months (with an average of 7.2 ± 3 months). The milk was prevented from cooling down and EV isolation started within 30 min after collection. Informed consent was signed by all donors and this study was approved by the local ethics committee.

### 1.2 Human milk EV isolation and EV‐depleted milk control isolation

Isolation of milk EVs was performed as previously described with some modifications for functional analysis (Zonneveld et al., 2014). Whole milk was centrifuged at 22°C for 10 min at 3000 g (Beckman Coulter Allegra X‐12R, Fullerton, CA, USA). After removal of the cream layer the harvested milk supernatant was centrifuged at 3000 g again at 22°C and stored at ‐80°C until further processing. Thawed supernatant was centrifuged in polyallomer SW40 tubes (Beckman Coulter) at 5000 g for 30 min at 4°C and subsequently at 10,000 g (Beckman Coulter Optima L‐90K with a SW40Ti rotor). Next, 6.5 ml aliquots of the 10,000 g supernatant were loaded on top of a 60%–10% iodixanol gradient (Optiprep, Progen Biotechnik GmbH, Heidelberg, Germany) in a SW40 tube. Gradients were ultracentrifuged at 192,000 g (Beckman Coulter Optima L‐90K with a SW40Ti rotor) for 15–18 h. Following this centrifugation, the resulting EV‐depleted milk supernatant (6.5 ml) left on top of the gradient was collected and loaded onto a new iodixanol gradient and ultracentrifuged once more and further processed identically to milk EV samples, to obtain a donor‐matched procedural milk control. The EV and EV‐depleted samples from the iodixanol gradients were collected in fractions of 500 μl and densities of 1.06‐1.19 g/ml were pooled, as these had a high expression of the EV‐associated marker CD9 in Western blot for the EV sample (data not shown). Iodixanol was removed from samples by size exclusion gel filtration using a 20 ml column (Bio‐Rad Laboratories, Hercules, CA, USA) packed with 15 ml Sephadex *g* 100 (Sigma‐Aldrich, St. Louis, MO, USA) and elutriating 24 fractions of 1 ml with phenol red free RPMI 1640, or DMEM medium (Gibco, Invitrogen, Carlsbad, CA, USA). Eluates 3–9 contained EVs as these had a high protein concentration and a high expression of the EV‐associated marker CD63 (data not shown). The EV‐containing eluates were pooled and supplemented with 10% heat‐inactivated foetal calf serum (FCS; Sigma‐Aldrich), or 0.1% BSA (Sigma‐Aldrich), 2 mM ultraglutamine (BioWhittaker, Lonza, Switzerland), and 100 IU/ml penicillin and 100 mg/ml streptomycin (Gibco). Samples were frozen at ‐80°C until use. The isolated milk EVs were characterized by Western blot for EV‐associated markers CD9, CD63, Flotillin‐1 and HSP70, as well as the non‐EV marker lactoferrin. Concentrations of EVs used for *in vitro* assays were within or below the physiological range, as the starting volume of the 10,000 g milk supernatant (6.5 ml) from which the milk EVs were isolated is in the range of the pooled eluates (7 ml). We have submitted all relevant data of our experiments to the EV‐TRACK knowledgebase (EV‐TRACK ID: EV200007) (Van Deun et al., 2017).

### 1.3 Nanoparticle tracking analysis

NTA measurements were performed with a NanoSight NS300 (Malvern Instruments Ltd, UK) equipped with a 405 nm laser, a high sensitivity sCMOS camera and a NanoSight Syringe Pump (Malvern Instruments Ltd, UK) for flow mode measurements. The NTA 3.2 software build 3.2.16 was used for data acquisition and processing. Each sample was diluted in low endotoxin PBS (1:100 for milk EVs, 1:10 EV‐depleted controls) to obtain a concentration in the range of 1*10^8^‐1*10^9^ particles/ml and particles in frame between 70 and 110. For each sample 3 captures of 60 s each were acquired with a syringe pump flow rate of 20 AU and temperature controlled at 20°C. Captures were obtained with camera level 13 and default combination of shutter and gain. Capture processing was performed with detection threshold 9, viscosity set to water (0,9cP) and automatic blur and minimum track length. The number of valid tracks in EV samples was always approximately 3 times greater than the recommended limit of 1000 (Gardiner et al., 2013).

### 1.4 SDS page and Western blotting

For characterization of samples during EV isolation, 100 μl aliquots were collected from iodixanol gradient Optiprep fractions, or eluates from the size exclusion gel filtration column. Isolated milk EVs, EV‐depleted sample, or fractions were pelleted by centrifugation for 65 min at 100,000 g (in a Beckman Coulter Optima Max‐XP with a TLA‐55 rotor) in polyallomer microcentrifuge tubes (Beckman). Pelleted fractions were resuspended in sample buffer (62.5 mM Tris pH 6.8, 2% SDS, 10% Glycerol), while pelleted EVs or EV‐depleted sample was resuspended in RIPA buffer (40 mM Tris pH 8.0, 150 mM NaCl, 1% Triton X‐100, 0.5% Na‐deoxycholate, 0.1% SDS). Whole cell lysates from Ca9‐22 cells were prepared from 5.2*10^5^ cells in 30 μl in RIPA buffer. Protein content of whole cell lysate was measured with the BCA protein assay (Pierce, Thermo Scientific, Landsmeer, Netherlands) to quantify the protein content of the samples in order to equalize for input material for SDS page. Samples were heated at 95°C for 3 min and run on an 8%–16% or 4%–20% TGX‐Criterion gel (Bio‐Rad). The separated proteins were transferred to PVDF membranes and blocked in PBS containing 0.2% fish skin gelatin (Sigma‐Aldrich) and 0.1% Tween‐20. Proteins were detected by immunoblotting using mouse anti‐human CD9 (clone HI9a, BioLegend, dilution 1:1000), mouse anti‐human CD63 (clone TS63, Abcam, dilution 1:1000), mouse anti‐human flotillin‐1 (clone 18, BD Biosciences, MA, USA, dilution 1:500, sample reduced with β‐mercaptoethanol) and mouse anti‐human HSC70/HSP70 (clone N27F3‐4, ENZO dilution 1:1000, sample reduced with β‐mercaptoethanol) and rabbit anti‐human lactoferrin (polyclonal, Abcam, dilution 1:5000), rabbit anti‐human TLR3 (D10F10, Cell Signaling TECHNOLOGY, dilution 1:1000, sample reduced with β‐mercaptoethanol), mouse anti‐human GAPDH (mAbcam 4984, Abcam, dilution 1:1000), mouse anti‐human CSTB (clone 225228, R&D Systems, dilution 1:500, sample reduced with β‐mercaptoethanol). Goat anti‐mouse‐HRP (Jackson Immuno Research, Suffolk, UK; dilution 1:10,000), or goat anti‐rabbit‐HRP (DAKO, dilution 1:1000) was used as secondary antibody. HRP conjugated antibodies were detected using SuperSignal West Dura Chemiluminescent Substrate (Thermo Scientific and ChemiDoc XRS and Image Lab 5.1 (Bio‐Rad).

### 1.5 Re‐epithelialization assay

Ca9‐22 (JCRB0625) gingival epithelial cells (JCRB Cell Bank, Osaka, JP) were cultured in DMEM containing Glutamax (Gibco) and supplemented with 10% FCS (Gibco), 100 U/ml penicillin and 100 μg/ml streptomycin (Sigma‐Aldrich). The re‐epithelialization assay was performed as previously described (Fernandez‐Gutierrez et al., 2017). For this, cells were seeded at 3.5 × 10^4^ cells/well in 96‐well flat bottom tissue culture treated plates (BD Falcon, Corning, NY, USA) and left to reattach and form a confluent monolayer overnight. Cells were starved in FCS‐free DMEM for 2 h prior to experiments and labelled during the last 20 min of starvation with 2 μM CellTracker Red CMTPX and 2 μg/ml Hoechst 33342 (both from Molecular Probes, Eugene, OR, USA). A gap was made in the cell monolayers of every well using an HTS Scratcher (Peira, Antwerp, BE). Cells were washed twice with PBS (Gibco) and cultured in FCS‐free DMEM as non‐treated control, 4 ng/ml human transforming growth factor α (TGFα; R&D Systems, MN, USA) as a positive control, or with 100 μl milk EVs, or 100 μl EV‐depleted control. For inhibition of migration via p38 MAPK or proliferation via MEK1/2 validated and selective inhibitors were used, respectively 10 μM of p38 inhibitor SB203580 (4‐(4‐fluorophenyl)‐2‐(4‐methylsulfinylphenyl)‐5‐(4‐pyridyl)‐imidazole (Cuenda et al., 1995; Kumar et al., 1999)) or 10 μM of MEK1/2 inhibitor U0126 (Favata et al., 1998) (both from Cell Signaling Technology, Danvers, MA, USA). Importantly, the concentrations used were in a range that would not induce off‐target side effects (Favata et al., 1998; Kumar et al., 1999). Cells were monitored by live cell imaging using the BD Pathway 855 Bioimaging System (BD Biosciences). Images of the same frame of each well were acquired every 20 min for 5 h or until the gaps in the positive controls were closed. Image segmentation and data analysis were carried out using CellProfiler 2.1.1 (https://www.cellprofiler.org/) and FCS Express 4 Plus (De Novo Software, Glendale, CA, USA). A nonlinear least squares regression was used to fit the modified Gompertz function (Fernandez‐Gutierrez et al., 2017) through the re‐epithelialization measurements obtained per well. The repair rate parameter derived from the model was used to calculate the average re‐epithelialization rate (cells/min) from technical triplicates. An integrated pipeline for image analysis, single cell enumeration, and kinetic modelling is available online through the Galaxy platform (Fernandez‐Gutierrez et al., 2018) and a detailed protocol of the assay can be found in Nature Protocol Exchange (Fernandez‐Gutierrez et al., 2020). To determine cell displacement, sequential images per treatment were loaded into Fiji (ImageJ), stacked (*z* as time), and converted into a hyperstack. The stacks were subsequently tracked using TrackMate (Tinevez et al., 2017) (v. 5.2.0). Cell nuclei were detected using LoG detector (estimated blob diameter 0.115, threshold 15, with sub‐pixel localization). All selected spots were used for further analysis (no thresholding) and spots were tracked using the linear motion LAP tracker (initial search radius 0.2, search radius 0.3, max frame gap 2). The tracks were pseudo‐coloured according to the total track displacement that was scaled for each individual image series. Feature tables for time and individual tracks as well as their corresponding cell displacement were exported. Subsequently, the tracks were visualized by capture overlay (HyperstackDisplayer) with their corresponding colours for total track displacement.

### 1.6 TLR reporter assay

HEK‐Blue‐hTLR2, HEK‐Blue‐hTLR3, HEK‐Blue‐hTLR4, and HEK‐Blue‐hTLR9 reporter cell lines (Invivogen, Toulouse, FR) were cultured in DMEM medium containing Glutamax (Gibco), supplemented with 8.5% heat‐inactivated FCS (Bodinco), 50 units/ml penicillin (Sigma‐Aldrich), 50 μg/ml streptomycin (Sigma‐Aldrich) and 100 μg/ml Normocin (Invivogen). Additionally, the HEK‐Blue hTLR9 culture medium was supplemented with 10 μg/ml blasticidin (Invivogen) and 100 μg/ml zeocin (Invivogen), the HEK‐Blue hTLR3 culture medium with 30 μg/ml blasticidin and 100 μg/ml zeocin and the HEK‐Blue hTLR4 and –hTLR2 culture medium with 1x HEk‐Blue Selection (Invivogen). For experiments, cells were cultured in 96‐well flat bottom tissue culture treated plates with 90 μl milk EVs or EV‐depleted with a final volume/well 110 μl. Agonists used were 100 ng/ml Pam3CSK, 5 μg/ml Poly I:C, 10 ng/ml LPS‐EK, or 1 μg/ml CpG ODN2006 (all from Invivogen). Cells were cultured for 16 h after which supernatant was harvested and SEAP reporter protein secretion was determined using QUANTI‐Blue detection medium (Invivogen) and measuring absorption at 650 nm on a 550 Microplate reader (Bio‐Rad) as per manufacturer's instructions.

### 1.7 TLR stimulation of Ca9‐22 cells

Ca9‐22 cells were seeded at 3.5 × 10^4^ cells/well in 96‐well flat bottom plates (Corning) with DMEM medium containing Glutamax (Gibco) and supplemented with 10% FCS (Gibco) (Sigma‐Aldrich), 100 U/ml penicillin and 100 μg/ml streptomycin (Sigma‐Aldrich). The next day, the medium was replaced by FCS‐free medium containing 0.1% BSA (Sigma‐Aldrich) and cells were stimulated with 100 μl milk EVs or EV‐depleted controls in the presence or absence of 5 μg/ml Poly I:C (Invivogen). After 4 or 5 h, cells were washed with PBS (Gibco), harvested and stored at ‐80°C in RLT buffer with β‐mercaptoethanol from the RNeasy Mini prep Kit (Qiagen, Hilden, Germany) according to manufacturer's instructions until cDNA synthesis for RT^2^ Profiler PCR array. For TLR3 and GAPDH Western blot, cells were stored in RIPA buffer (40 mM Tris pH 8.0, 150 mM NaCl, 1% Triton X‐100, 0.5% Na‐deoxycholate, 0.1% SDS composition) buffer until use.

### 1.8 Endotoxin quantitation

The gram‐negative bacterial endotoxin concentration in the EV and EV‐depleted samples was measured using the Pierce LAL Chromogenic Endotoxin Quantitation Kit (ThermoFisher, MA, USA) according to the manufacturer's instructions.

### 1.9 RT‐qPCR and RT^2^ Profiler PCR array

Total RNA was extracted from Ca9‐22 cells after 4 or 5 h of culture using the RNeasy Mini prep Kit and cDNA was prepared using the RT^2^ First Strand Kit (Qiagen) or the qScript cDNA Synthesis Kit (Quanta Biosciences) according to the manufacturer's instructions. Real‐time quantitative PCR (RT‐qPCR) was performed on a Rotor‐Gene Q2plex real‐time cycler (Qiagen). For relative gene expressions of *IL6* and *CXCL8*, delta Ct‐values were log‐transformed with *ACTB* and *GAPDH* as internal controls. For gene expression profiling, cDNA was added to the Human Toll‐like receptor signalling pathway RT^2^ Profiler PCR array (Qiagen) and run on an iCycler MyiQ (Bio‐Rad). The relative expression levels of each gene were normalized to the expression level of 5 reference genes included in the array (*ACTB, B2M, GAPDH, HPRT1 and RPLP0*). Delta Ct‐values were log‐transformed and analyzed by the web‐based software GeneGlobe Data Analysis Center (Qiagen). Genes for which gene expression levels had a Ct > 35 in all test conditions were excluded from analysis.

### 1.10 CD4+  T cell isolation

Human peripheral blood mononuclear cells (PBMC) were isolated from Buffy coats by Lymphoprep density gradient centrifugation (Axis‐shield, Dundee, United Kingdom and Nycomed, Zurich, Switzerland). PBMC were cultured in RPMI 1640 supplemented with Glutamax and sodium pyruvate (Gibco), 2.5% FCS and 100 IU/ml penicillin and 100 mg/ml streptomycin (Gibco). CD4+ T cells were isolated from PBMC using CD4+ T cell isolation kit (Miltenyi Biotec, Bergisch Gladbach, Germany) according to manufacturer's instructions (average purity of 95%) and were used directly in experiments or fractionated into CD45RA+ and CD45RO+ subsets using anti‐CD45RA‐PE (UCHL‐1, Dako) and anti‐PE magnetic beads (Miltenyi Biotec) resulting in > 97% pure T cell subpopulations.

### 1.11 T cell stimulation

PBMCs were seeded at 4 × 10^6^ cells/ml in 12 wells plates (Corning) coated with 1.5 μg/ml αCD3 (CLB‐T3/4.E, 1XE from Sanquin, Amsterdam, The Netherlands) and cultured in RPMI 1640 medium (Gibco) with 10% FCS (Sigma‐Aldrich), 750 μl milk EVs or EV‐depleted controls in a total volume of 1 ml and cultured for 6 days. Purified CD4+ T cells were seeded at 0.5 × 10^6^ cells/ml in 48 wells plates (Corning) coated with 0.5 – 1.5 μg/ml αCD3 (CLB‐T3/4.E, 1XE from Sanquin, Amsterdam, The Netherlands) and cultured with 70 ng/ml – 1 μg/ml soluble αCD28 (CLB‐CD28/1, 15E8 from Sanquin), RPMI 1640 medium (Gibco) with 10% FCS (Sigma‐Aldrich), 750 μl milk EVs or EV‐depleted controls in a total volume of 1 ml and cultured for the indicated amount of time. Notably, the final concentration of milk EVs was 75% of the physiological concentration of the EV preparations as obtained after SEC when 750 μl of milk EVs were added. Hence, formally T cell modulation studies were performed with a sub physiological concentration. Additionally, lower volumes of milk EV samples were added to determine dose‐dependent effects and add back experiments were performed in which milk EVs were added to the EV‐depleted milk control in order to recover EV‐mediated effects.

### 1.12 Multiplex cytokine analysis

Supernatants of stimulated T cells were harvested on day 6 of culture and analyzed using the LEGENDplex human Thelper 1/2/9/17 multiplex kit (BioLegend). Beads were acquired on a BD Canto II (BD Biosciences) and analyzed with LEGENDplex V7.0.

### 1.13 T cell suppression assay

T cell suppression assay was performed as described previously (Van Der Aar et al., 2011). In brief, CD4^+^CD45RA^+^ T cells were activated for 5–6 days with 1.5 μg/ml plate bound αCD3 (Sanquin) and 1 μg/ml soluble αCD28 (Sanquin) in medium, or in the presence of EV or EV‐depleted controls. T cells were subsequently harvested, irradiated (3,000 rad), washed and counted. Cells were replated at a 2:1 ratio with 0.5 μM CFSE (Sigma‐Aldrich) labelled autologous CD4^+^CD45RO^+^ responder cells in the presence of monocyte‐derived allogeneic DC. Cells were incubated for 5–6 days and CFSE dilution of T cells was determined on a BD Canto II (BD Biosciences).

### 1.14 Restimulation assay

10 × 10^6^ cells/ml CD4^+^CD45RA^+^ T cells were cultured for 3 days in the presence of 1.5 μg/ml plate bound αCD3 (CLB‐T3/4.E, 1XE Sanquin) and 1 μg/ml soluble αCD28 (CLB‐CD28/1, 15E8 Sanquin) in the presence or absence of milk EVs or EV depleted control. T cells were subsequently harvested, washed, counted and reseeded at a 4:1 ratio with allogeneic monocyte‐derived DC generated as previously described (De Jong et al., 2002) in a mixed lymphocyte response. Cells were incubated for 3 days total and to determine T cell proliferation 11 KBQ/well ^3^H‐thymidine (Radiochemical Center, Amersham, Little Chalfont, UK) was added. The incorporated ^3^H‐thymidine was measured after 16 h by liquid scintillation spectroscopy.

### 1.15 Flow cytometry

Expression of E‐cadherin and EPCAM was determined after Ca9‐22 gingival epithelial cells were cultured in DMEM containing Glutamax (Gibco), 100 U/ml penicillin and 100 μg/ml streptomycin (Sigma‐Aldrich). Cells were seeded at 3.5 × 10^4^ cells/well in 96‐well flat bottom tissue culture treated plates (BD Falcon) and left to reattach and form a confluent monolayer overnight. Cells were starved in FCS‐free DMEM for 2 h prior to experiments after which they were washed twice with PBS (Gibco) and cultured in FCS‐free DMEM, 100 μl milk EVs, or 100 μl EV‐depleted control for 5 h. Following culture, cells were harvested and stained with fluorescent conjugated antibodies, which were E‐cadherin‐PE (clone 67A4, BD Biosciences, dilution 1:50) and EPCAM‐APC (EBA1, BD Biosciences, dilution 1:50), or with corresponding isotypes which were PE mouse IgG1 (BD Biosciences, dilution 1:50) or APC mouse IgG1 (BD Biosciences, dilution 1:50). The gating strategy for the characterization of E‐cadherin and EPCAM on epithelial cells is indicated in Supplementary Figure 3. Proliferation of purified CD4+ T cells was assessed by labelling cells with 2 μM CellTrace Violet (Invitrogen) or 0.5 μM CFSE (Sigma‐Aldrich) prior to culture. Following culture, cells were harvested and stained with fluorescent conjugated antibodies. Antibodies used were: CD3‐PE‐Cy7 (UCHT1, dilution 1:100), CD4‐PerCP‐Cy5.5 (OKT4, dilution 1:50), CD25‐Alexa488 (BC96, dilution 1:20), CD45RA‐PE (HI100, dilution 1:50), and CD45RO‐APC‐Cy7 (UCHL1, dilution 1:50), CD127‐PE‐Cy7 (A019D5, dilution 1:20) (all from BioLegend). FoxP3‐APC (eBioscience, San Diego, CA, USA, 236A/E7, dilution 1:25). Intracellular staining for Treg phenotype were done using the Anti‐human Foxp3 Staining Set (eBioscience) according to the manufacturer's protocol. The gating strategy for the characterization Tregs is indicated in Supplementary Figure 12. Cells were measured on a BD Canto II (BD Biosciences), and analyzed by FlowJo (v10.1 FlowJo, Ashland, OR, USA).

### 1.16 Functional enrichment, network‐ and annotation analysis

Previously, we unravelled the common milk‐EV proteome (van Herwijnen et al., 2016), in which 367 proteins were identified in all 7 human milk EV samples tested. These proteins were loaded into the STRING (version 10.5) protein‐protein interaction network analysis tool (Szklarczyk et al., 2015), of which 362 proteins matched the STRING database (Supplementary File 3). Enrichment analysis for biological process on these proteins identified n = 784 significantly enriched GO‐terms, of which several linked to the observed *in vitro* effects of milk EVs. GO‐terms were selected that linked to cell cycle (proliferation) and regulation of migration (n = 33), or regulation of TLR signalling (n = 26), or regulation of T cell activation (n = 48) (Supplementary File 3 for data and complete workflow). As enrichment analysis alone does not provide information on the molecular action of a protein, or its position in a signalling cascade, we next performed a comprehensive functional annotation analysis. For that purpose, signalling pathways underlying the observed *in vitro* effects were constructed using Uniprot entries and the associated sources from Uniprot itself for each individual selected milk EV protein from the enrichment analysis. This pathway analysis was supplemented with a general literature search on both the individual milk EV proteins, as well as the signalling pathways associated to the *in vitro* observations in order to prevent biased results solely from the enrichment data. Annotation analysis on the selected milk EV proteins was done to determine their interaction with other proteins (either given a common gene name, or a synonym when widely used in literature) and the type of interaction (activating or inhibiting). For each milk EV protein it was decided whether the protein itself, or its cellular interaction partner was relevant to signalling pathways involved in the particular *in vitro* experiments. Although we could link many milk EV proteins to underlying signalling cascades, yet for some proteins there was insufficient experimental evidence to do so (Supplementary File 3). In the end, all relevant signalling cascades from the full functional annotation analysis are depicted in supplementary figures (Supplementary Figure 2B for proliferation and migration; Supplementary Figure 5B for TLR signalling; Supplementary Figure 13B for T cell activation), in which ‘hot spots’ were defined as a node in the signalling cascade where a cellular protein would have at least 6 potential interaction partners. Key pathways from the full annotation analysis are shown as a summary in the main figures whereas irrelevant signalling cascades are omitted for clarity. Additionally, protein‐protein network analysis on the identified milk EV proteins that linked to the selected GO‐terms was performed in STRING (minimum required interaction score set to high confidence 0.700), followed by k‐means clustering in order to construct clusters of interaction proteins within the network.

### 1.17 Statistical analysis

Data normality was assessed by Shapiro‐Wilks test. Normal distributed data were analyzed by paired t‐test or one‐way ANOVA with Tukey's or Sidak's multiple comparison test. Data that did not have homogenous variances were alternatively tested with Brown‐Forsythe and Welch ANOVA and Dunnett's T3 multiple comparison test or Games‐Howell's correction, depending on sample size. Figure 3a‐d were log‐transformed and analyzed in a 2‐way mixed model (R‐Package lme4 V1.1‐26) having EV donor and experiment number as random factors with post‐hoc analysis of the least‐squares means and performed with Bonferroni multiple comparison correction. Non‐normally distributed data were analyzed by Wilcoxon matched‐pairs signed rank test or Kruskal‐Wallis test with Dunn's multiple comparisons test. GO‐analysis in String was tested with a Fisher's exact test followed by a correction for multiple testing. Analyses were performed in GraphPad Prism Software V8.3.1. and R V4.0.3. Significance was defined as *P < 0.05; **P < 0.01; ***P < 0.001 and **** *P* < 0.0001.

## AUTHOR CONTRIBUTIONS

Marca H.M. Wauben, Esther N.M. Nolte‐’t Hoen and Frank A. Redegeld conceived and designed the study. Marijke I. Zonneveld, Martijn J.C. van Herwijnen, Marcela M. Fernandez‐Gutierrez, Alberta Giovanazzi, Anne Marit de Groot, Marije Kleinjan and Toni M.M. van Capel performed experiments. Marijke I. Zonneveld, Martijn J.C. van Herwijnen, Marcela M. Fernandez‐Gutierrez, Alberta Giovanazzi, Anne Marit de Groot, Marije Kleinjan, and Marca H.M. Wauben analyzed the data. Marijke I. Zonneveld, Martijn J.C. van Herwijnen, Marcela M. Fernandez‐Gutierrez, Anne Marit de Groot, Alice J.A.M. Sijts, Leonie S. Taams, Esther C. de Jong, Marije Kleinjan, Esther N.M. Nolte‐’t Hoen, Marca H.M. Wauben wrote and revised the manuscript. All authors contributed to editing the paper.

## Supporting information

Supplementary information

Supplementary information

Supplementary information

Supplementary information

Supplementary information
