# Supplementary material for: Human milk extracellular vesicles target nodes in interconnected signalling pathways that enhance oral epithelial barrier function and dampen immune responses
Source: J Extracell Vesicles. 2021 Mar 10;10(5):e12071. doi: 10.1002/jev2.12071 (PMC7944547; doi:10.1002/jev2.12071)
Supplement: Supplementary file 1 — Supplementary information [file JEV2-10-e12071-s005.docx]

**Supplementary Figures 1-14**

**
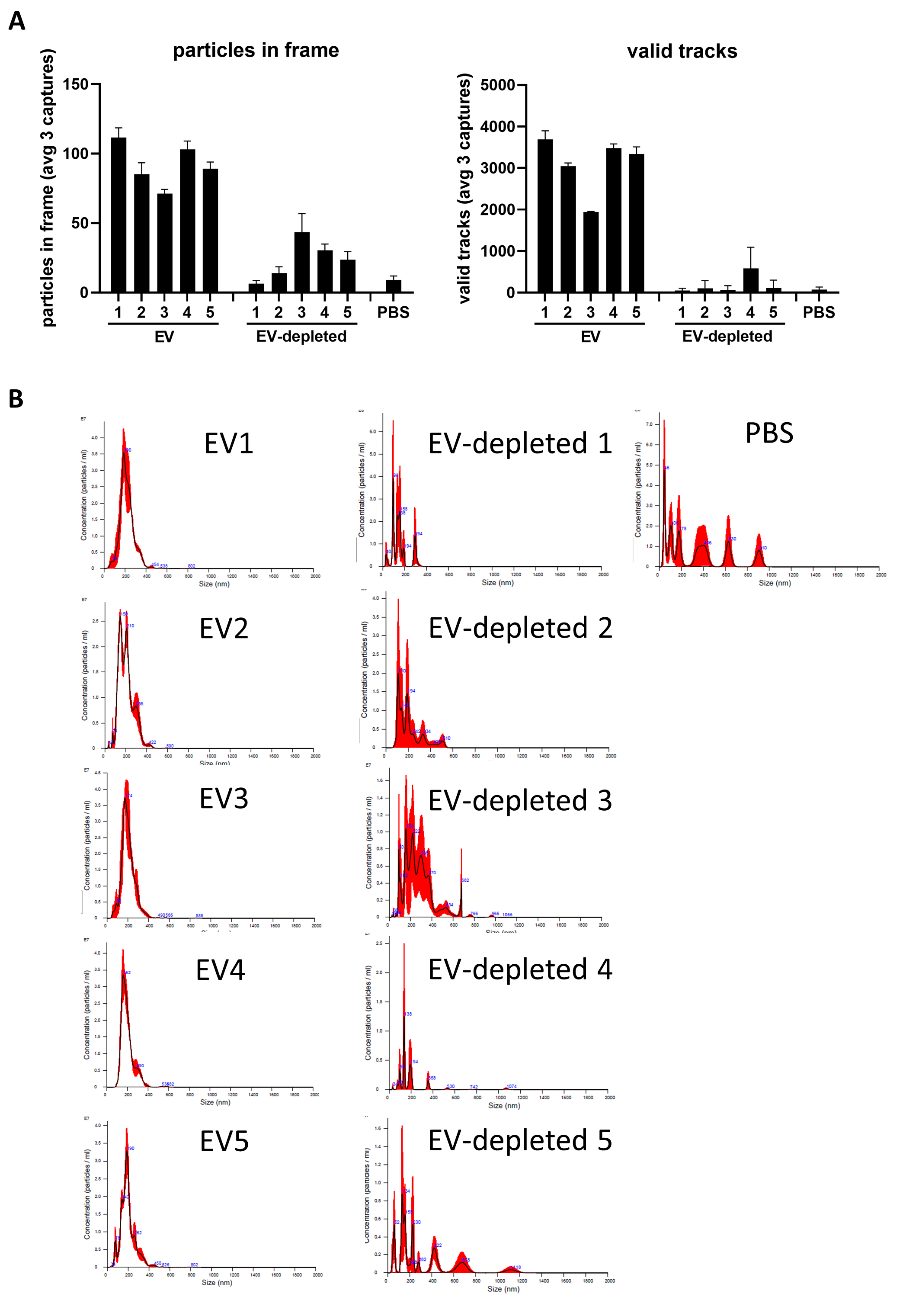
**

**Supplementary Fig. 1. NTA measurements**

A) Values of particles in frame of each EV sample, EV-depleted control, or PBS. Only measurements with particles/frame rate in the range of 70-110 corresponding to the recommended concentration 1*10^8^-1*10^9^ particles/ml were further analyzed. Data are from triplicate 60-sec captures. B) Values of valid tracks for Particle Size Distribution by NTA. Only measurements with at least 1000 valid tracks were further analyzed. EV-depleted controls did not meet both criteria due to low number of particles present in the samples resulting in the detection of high background noise. In red the SD is shown from the triplicate 60-sec captures. See Supplementary File 1 for representative videos of a milk EV sample and an EV-depleted procedural control sample.

­­­­­­­­­­­­­­­­­­­­­­­­_________________________________________________________________________________________________________________________________________________________________________________________________________________________________________________________________________________________________________________________


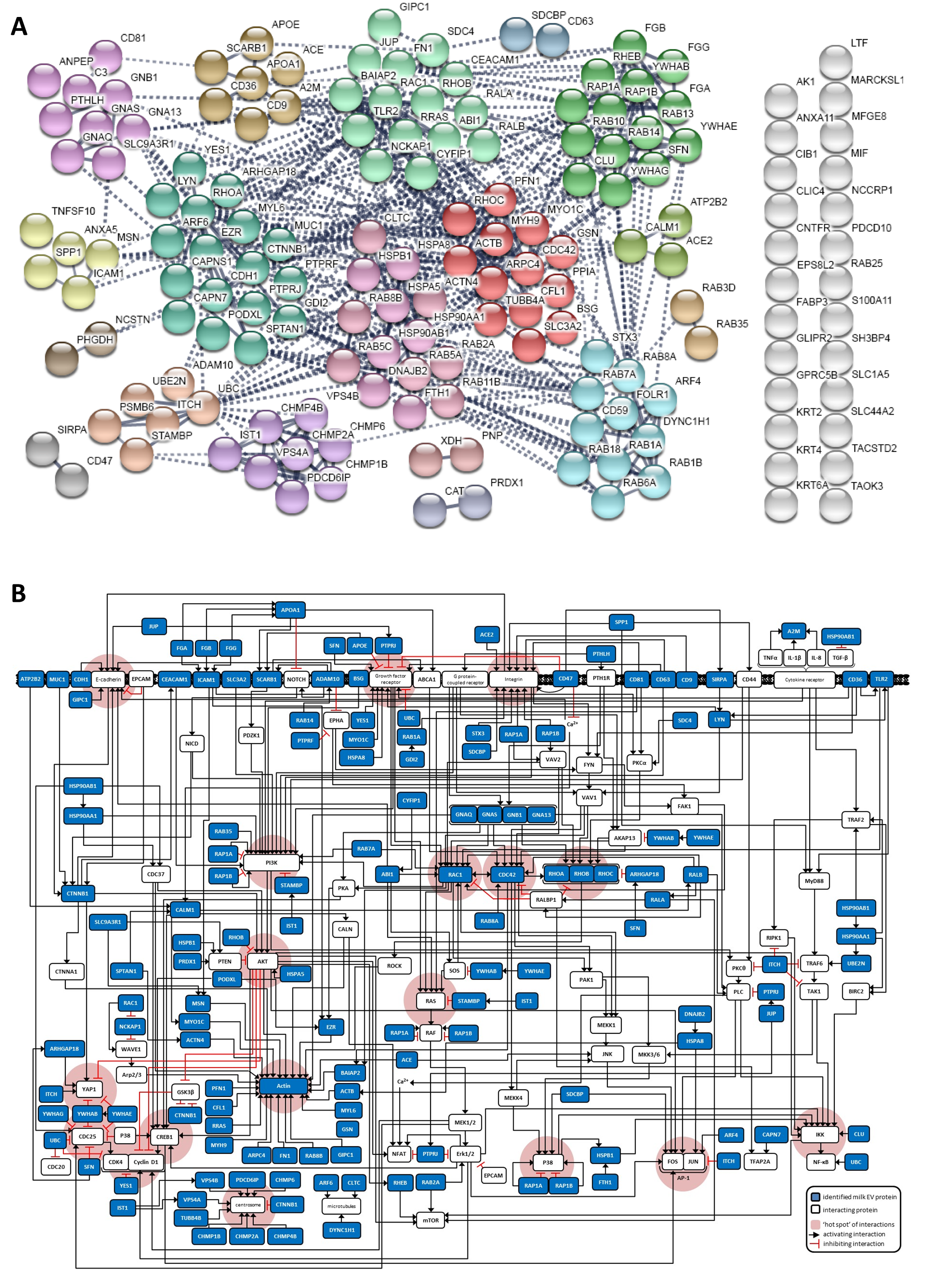


**Supplementary Fig. 2: Enrichment and protein-protein network analysis allowed complete integration of human milk EV proteins into signaling pathways associated to cell cycle (proliferation) and migration and revealed interactions at multiple levels in the signaling cascades.**

A) Protein-protein interaction analysis on the identified milk EV proteins that linked to the selected GO-terms was performed (minimum required interaction score set to high confidence 0.700), followed by k-means clustering (showing 18 clusters) in order to visualize the most likely occurring clusters within the network. A total of 134 proteins formed protein-protein interactions (which is 84.3%) and 25 proteins had no interaction with any other milk EV protein (which is 15.7%). Only those proteins that were part of an interaction network were further investigated for validated links to proliferation or migration signaling cascades. B) Functional annotation analysis of milk EV protein clusters that link to cell cycle (proliferation) and migration. The interaction of selected milk EV proteins (in blue) and cellular proteins (in white; either shown with their common gene name, or a synonym when widely used in literature) and the type of interaction (activating or inhibiting) within relevant signaling pathways are shown. If a protein has interactions with ≥ 6 other proteins, this node is shown in red as a ‘hotspot’. Although milk EV proteins were selected via relevant GO-terms, some proteins could not be linked to the specific signaling cascades and are not shown in B (see Supplementary File 3 for a full overview of the analysis).

_________________________________________________________________________________________________________________________________________________________________________________________________________________________________________________________________________________________________________________________


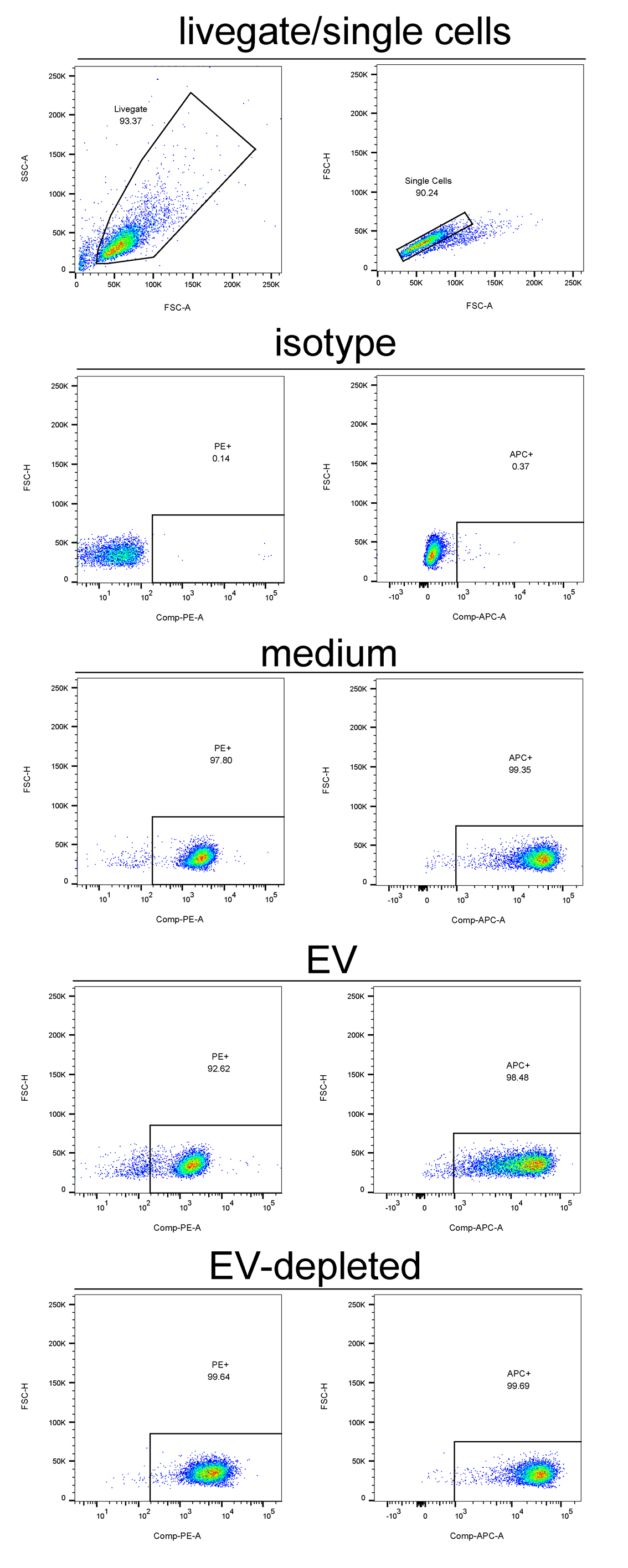


­­­­­­­­­­­­­­­­­­­­­­­­**Supplementary Fig. 3. Gating strategy for the characterization of E-cadherin+ or EPCAM+ epithelial cells**

Ca9-22 epithelial cells were cultured for 5 hours in medium, or together with milk EVs or EV-depleted control after which cell surface expression of E-cadherin or EPCAM was determined. First, a live gate was used to discriminate apoptotic cells and debris from live cells. Within the live gate, single cells were selected, and isotype controls were used to set gates to select PE+ (E-cadherin) or APC+ (EPCAM) cells. Dot plots shown are representative for each culture condition and all donors tested.

­­­­­­­­­­­­­­­­­­­­­­­­_________________________________________________________________________________________________________________________________________________________________________________________________________________________________________________________________________________________________________________________


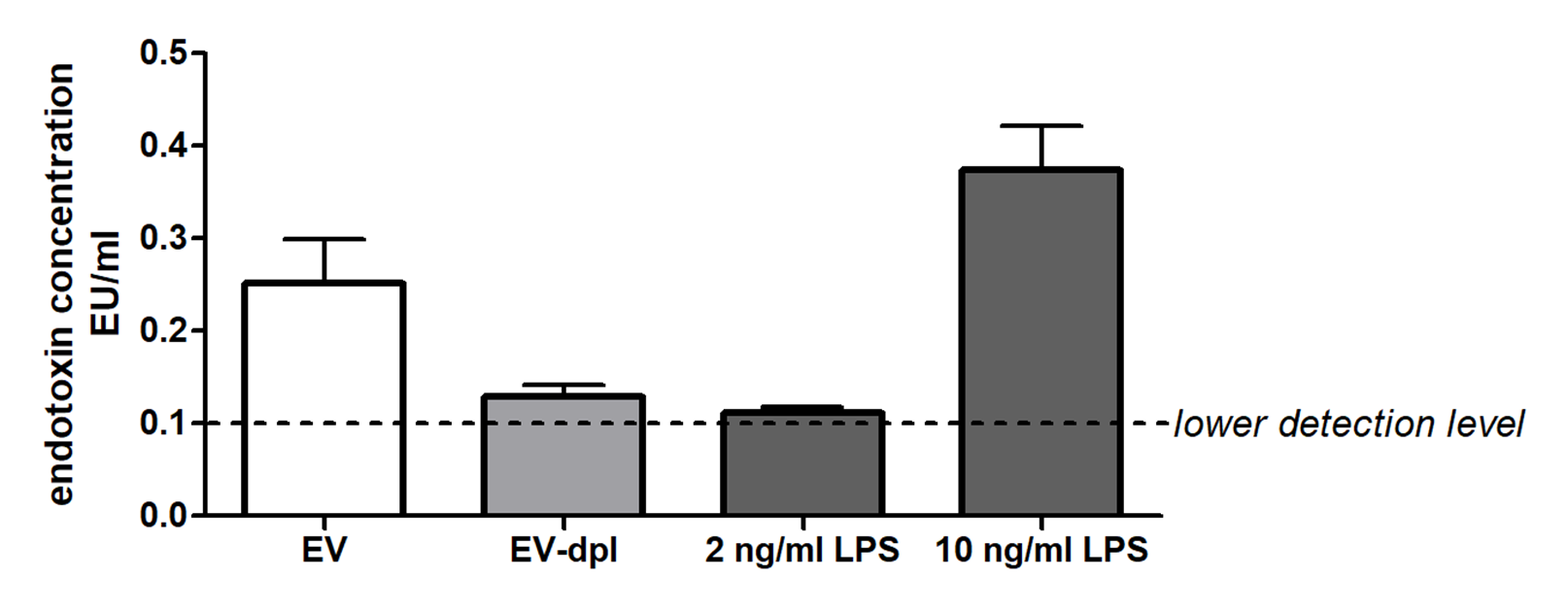


**Supplementary Fig. 4: Endotoxin levels in milk EVs or EV-depleted samples.**

Endotoxin levels were determined in milk EV samples (n=3) or EV-depleted control (n=2) using a LAL assay and compared to the positive control, which was LPS. As a reference the same concentration of LPS (10ng/mL) was used as in the TLR4 assay presented in Fig. 3B.

_________________________________________________________________________________________________________________________________________________________________________________________________________________________________________________________________________________________________________________________


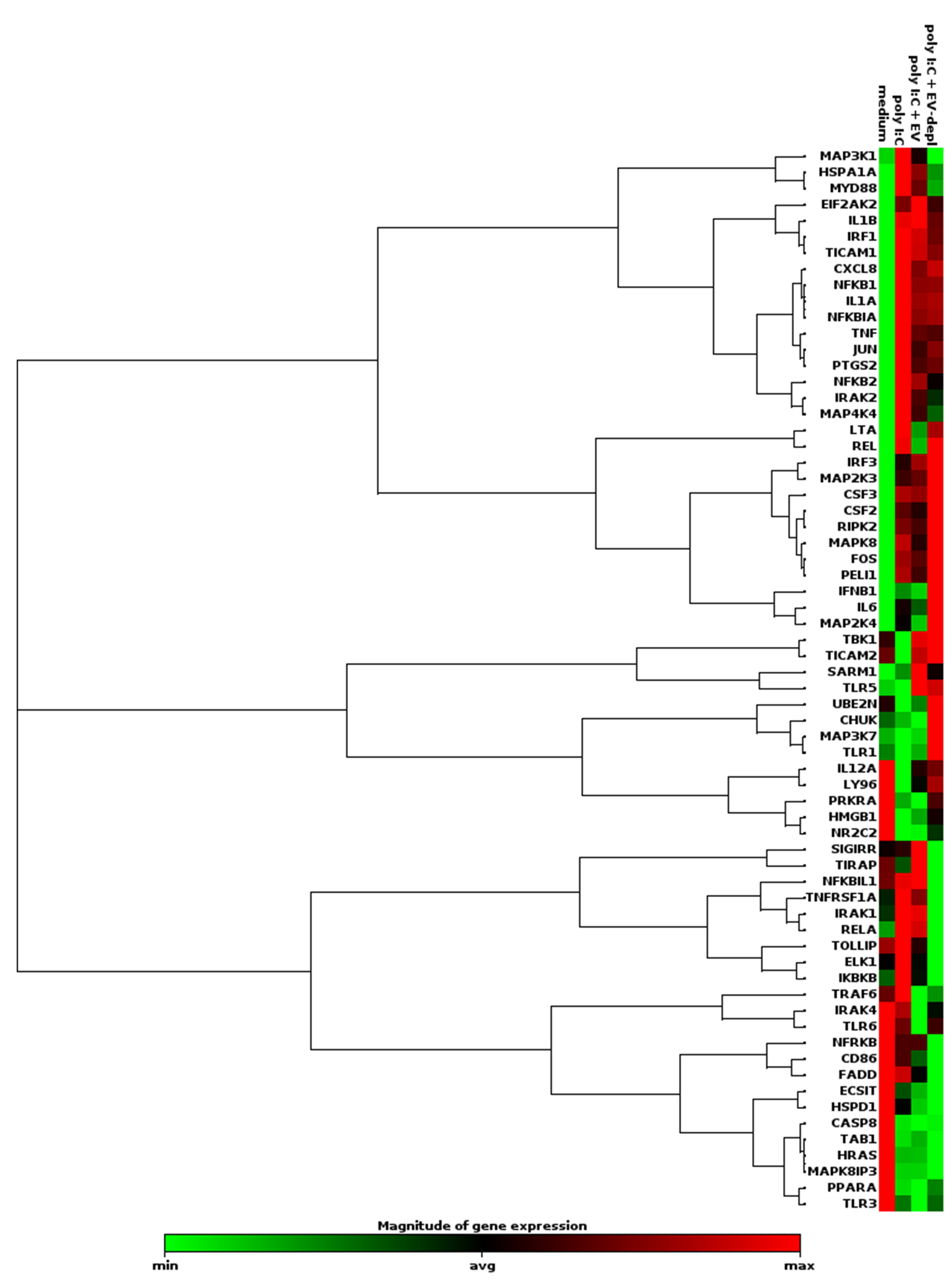


**Supplementary Fig. 5: Complete heatmap of cellular gene expression profile from gingival epithelial cells exposed to human milk EVs.**

Complete dataset from gene array in Fig. 3F, which shows the non-supervised hierarchical clustering of all culture conditions to display a heat map with dendrograms indicating co-regulated genes across groups or individual samples. The heat map of gene expression is shown as a gradient running from minimal gene expression (green) to maximal expression (red) for each gene analyzed. Genes for which gene expression levels had a Ct>35 in all test conditions were excluded from the analysis and household genes are not shown. Data is from 1 technical replicate derived from 1 experiment, with 1 milk donor.

­­­­­­­­­­­­­­­­­­­­­­­­_________________________________________________________________________________________________________________________________________________________________________________________________________________________________________________________________________________________________________________________


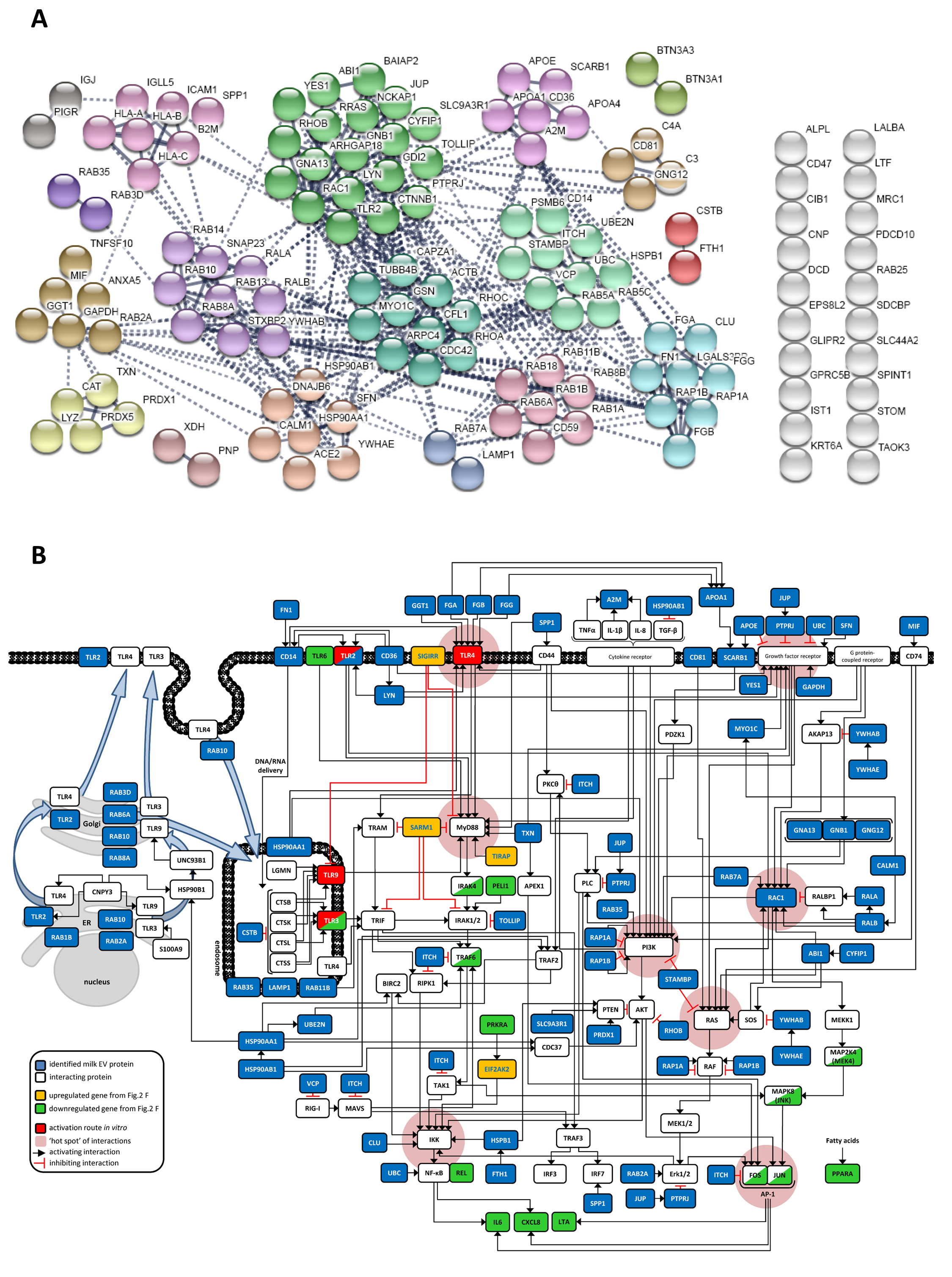


**Supplementary Fig. 6: Enrichment and protein-protein network analysis allowed complete integration of human milk EV proteins into signaling pathways associated to TLR signaling and revealed interactions at multiple levels in the signaling cascades.**

A) Protein-protein interaction analysis on the identified milk EV proteins that linked to the selected GO-terms was performed (minimum required interaction score set to high confidence 0.700), followed by k-means clustering (showing 18 clusters) in order to visualize the most likely occurring clusters within the network. A total of 110 proteins formed protein-protein interactions (which is 84.6%) and 20 proteins had no interaction with any other milk EV protein (which is 15.4%). Only those proteins that were part of an interaction network were further investigated for validated links to TLR signaling cascades. B) Functional annotation analysis of milk EV protein clusters that link to TLR signaling. The interaction of selected milk EV proteins (in blue) and cellular proteins (in white; either shown with their common gene name, or a synonym when widely used in literature) and the type of interaction (activating or inhibiting) within relevant signaling pathways are shown. Furthermore, we included in the model the TLR-associated genes (Fig. 3F) that were differentially expressed after incubation of the epithelial cells with TLR3 agonist in presence of milk EVs which resulted in either upregulated or downregulated gene expression compared to medium, agonist, or EV-depleted control. If a protein has interactions with ≥ 6 other proteins, this node is shown in red as a ‘hotspot’. Although milk EV proteins were selected via relevant GO-terms, some proteins could not be linked to the specific signaling cascades and are not shown in B (see Supplementary File 3 for a full overview of the analysis).

­­­­­­­­­­­­­­­­­­­­­­­­_________________________________________________________________________________________________________________________________________________________________________________________________________________________________________________________________________________________________________________________


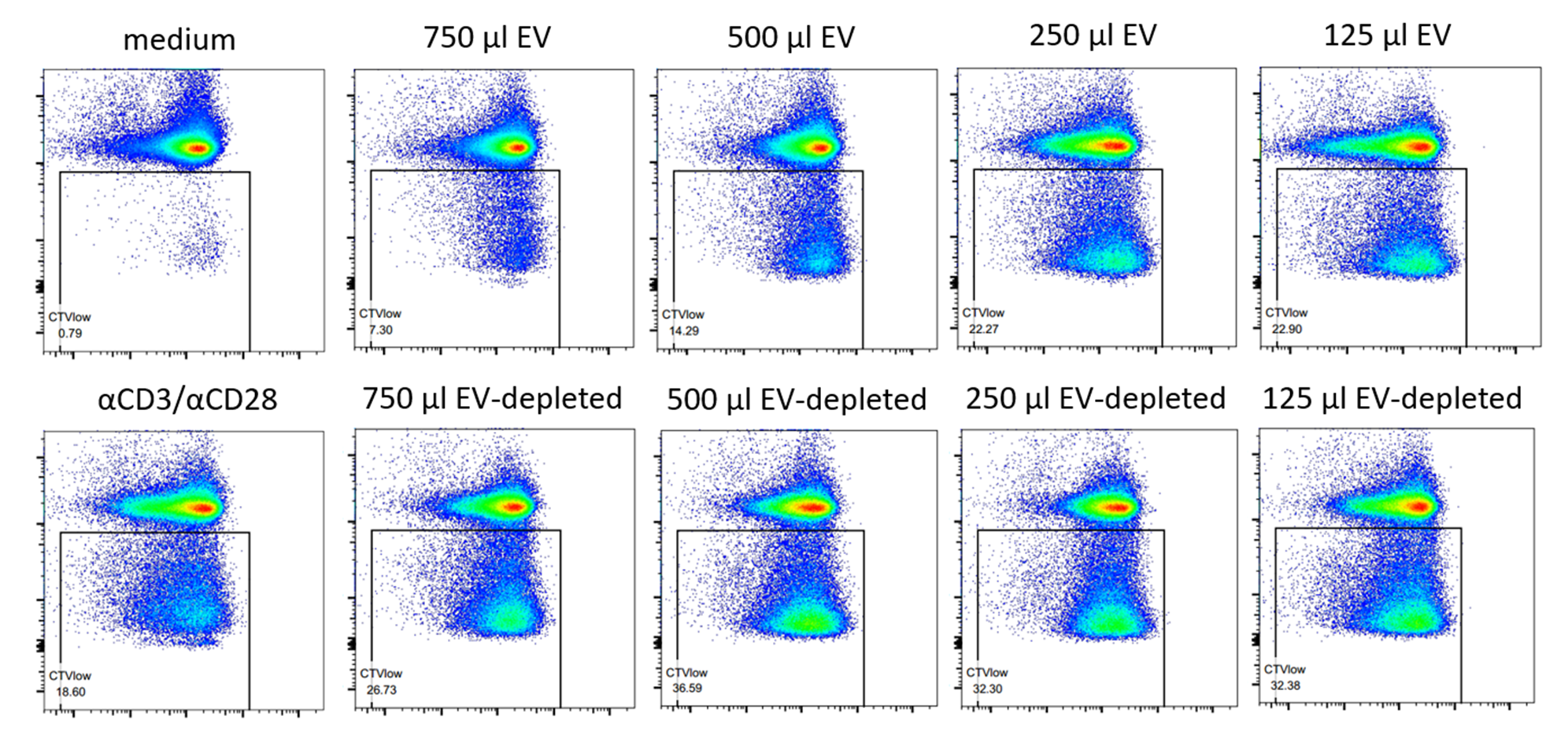


**Supplementary Fig. 7: Human milk EVs inhibit CD4+ T cell activation in a dose-dependent manner.**

Purified CD4+ T cells were labeled with Cell Trace Violet (CTV) and incubated in medium alone, or in the presence of αCD3 and αCD28 (αCD3/αCD28), or αCD3/αCD28 in the presence of EV or EV-depleted control for 6 days. Representative dot plots of CTV dilution are shown in response to the indicated conditions. Percentage CTVlow cells (gate) is expressed as a fraction of total CD4+ T cells. Data are representative of a single experiment using n=1 T cell donor and n=3 different milk donors.

_________________________________________________________________________________________________________________________________________________________________________________________________________________________________________________________________________________________________________________________


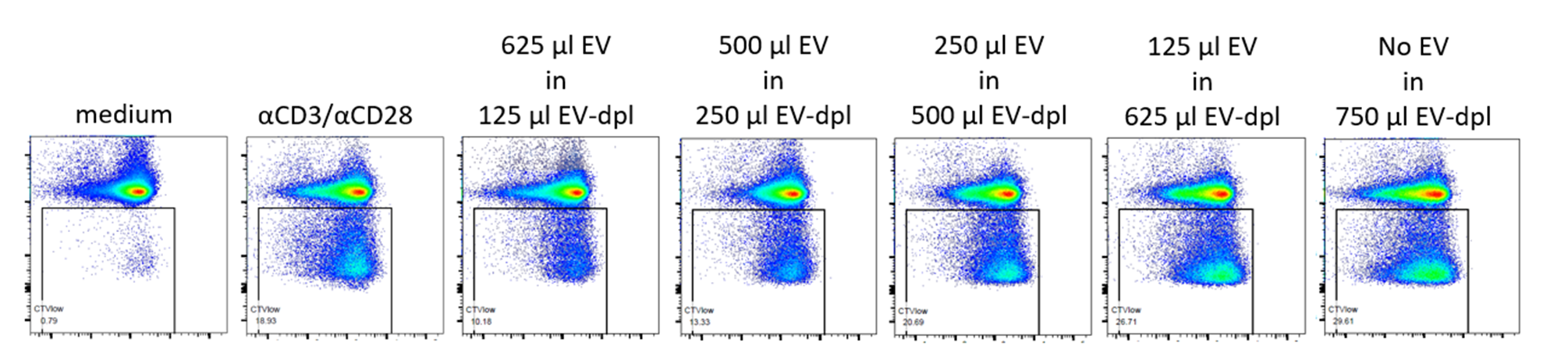


**Supplementary Fig. 8: Recovery of the lack of inhibitory capacity of the EV-depleted control after addition of milk EVs.**

Purified CD4+ T cells were labeled with Cell Trace Violet (CTV) and incubated in medium alone, or in the presence of αCD3 and αCD28 (αCD3/αCD28), or αCD3/αCD28 in the presence of EV or EV-depleted control for 6 days. Representative dot plots of CTV dilution are shown in response to the indicated conditions. Percentage CTVlow cells (gate) is expressed as a fraction of total CD4+ T cells. Data are representative of a single experiment using n=1 T cell donor and n=3 different milk donors.

­­­­­­­­­­­­­­­­­­­­­­­­_________________________________________________________________________________________________________________________________________________________________________________________________________________________________________________________________________________________________________________________


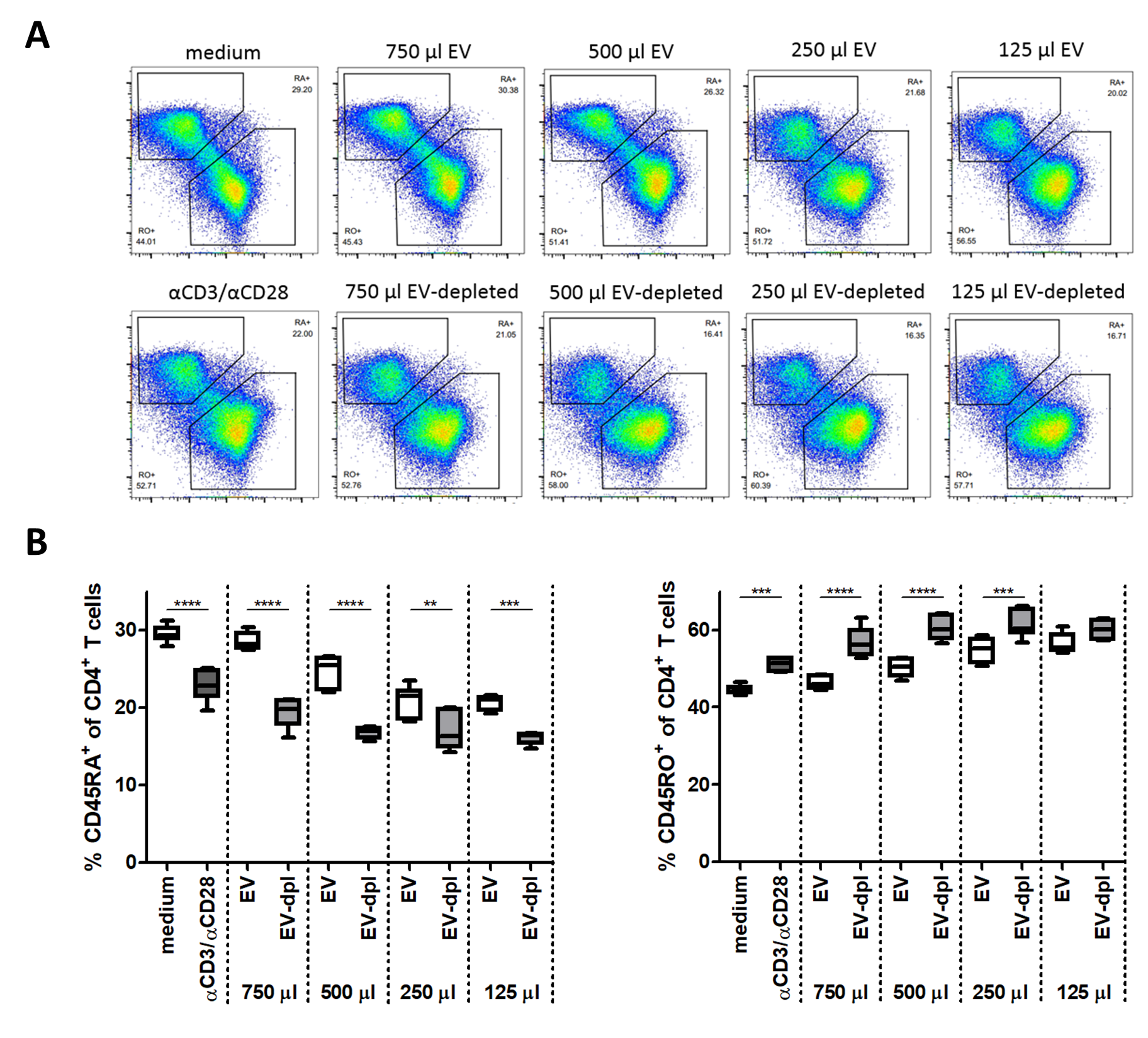


**Supplementary Fig. 9: Human milk EVs retain CD4+ T cells in a naïve phenotype in a dose-dependent manner.**

Purified CD4+ T cells were incubated in medium alone, or in the presence of αCD3 and αCD28 (αCD3/αCD28), or αCD3/αCD28 in the presence of EV or EV-depleted control for 6 days. A) Representative dot plots of CD4+ T cells stained for CD45RA and CD45RO after culture in the indicated conditions. Percentage of CD45RA+/CD45RO- and CD45RO+/CD45RA- cells in gates are expressed as a fraction of total CD4+ T cells. B and C) Quantification of the percentage CD45RA+ and CD45RO+ cells of total CD4 T cells following incubation with the indicated conditions. Box and whisker plots contain data of a single experiment using n=1 T cell donor and n=3 different milk donors. Significance was calculated with one-way ANOVA and Sidak’s multiple comparisons test and significance defined as ** p < 0.01; *** p < 0.001 and **** p < 0.001.

_________________________________________________________________________________________________________________________________________________________________________________________________________________________________________________________________________________________________________________________


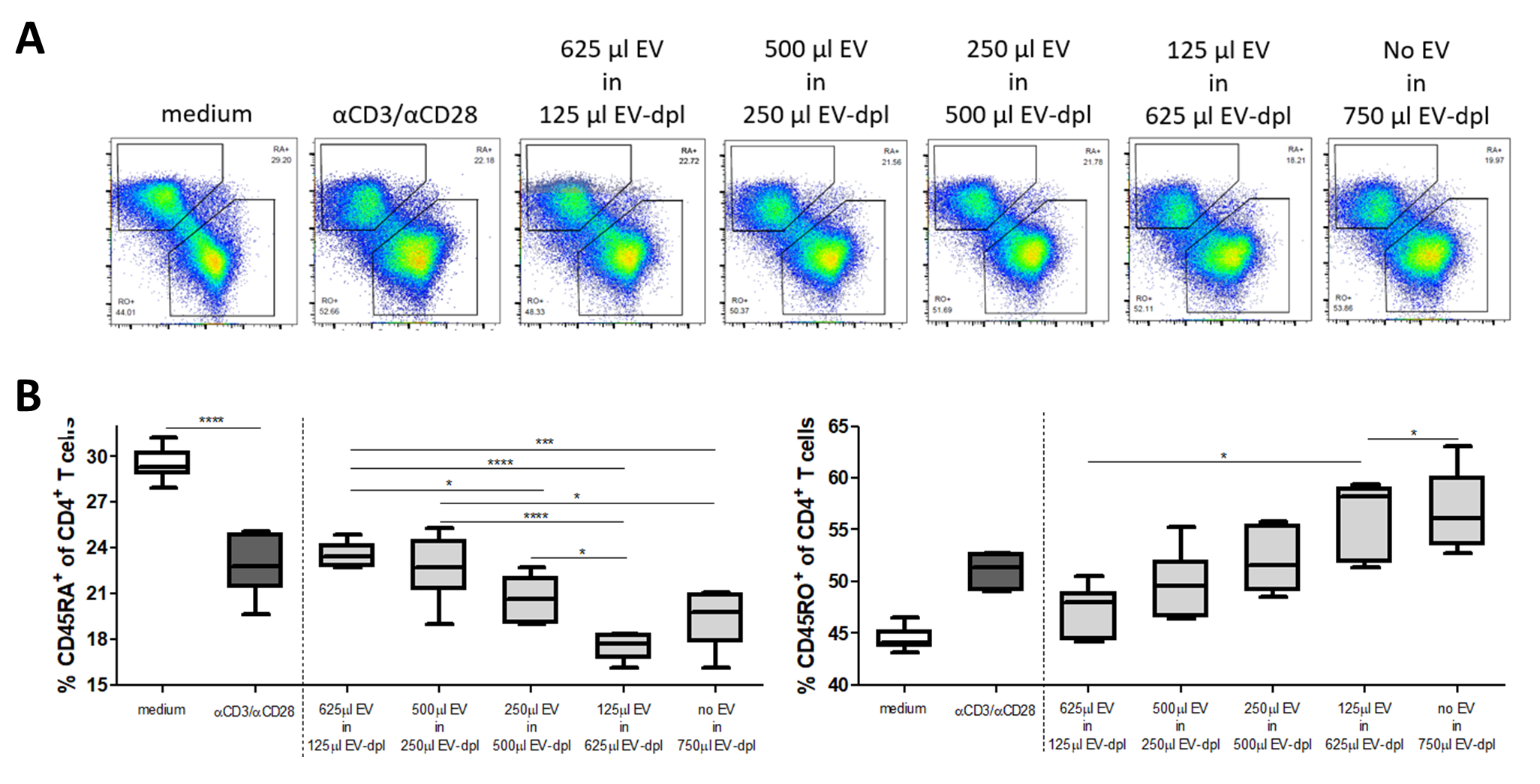


**Supplementary Fig. 10: Human milk EVs rescue the EV-depleted control in retaining CD4+ T cells in a naïve phenotype.**

Purified CD4+ T cells were incubated in medium alone, or in the presence of αCD3 and αCD28 (αCD3/αCD28), or αCD3/αCD28 in the presence of EV or EV-depleted control for 6 days. A) Representative dot plots of CD4+ T cells stained for CD45RA and CD45RO after culture in the indicated conditions. Percentage of CD45RA+/CD45RO- and CD45RO+/CD45RA- cells in gates are expressed as a fraction of total CD4+ T cells. B) Quantification of the percentage CD45RA+ and CD45RO+ cells of total CD4 T cells following incubation with the indicated conditions. Not that the origin of the y-axis does not start at 0. Significance was calculated with one-way ANOVA and Tukey’s multiple comparisons test (CD45RA) or Kruskal-Wallis and Dunn’s multiple comparison test (CD45RO) and significance defined as * p < 0.05; *** p < 0.001 and **** p < 0.0001.

_________________________________________________________________________________________________________________________________________________________________________________________________________________________________________________________________________________________________________________________


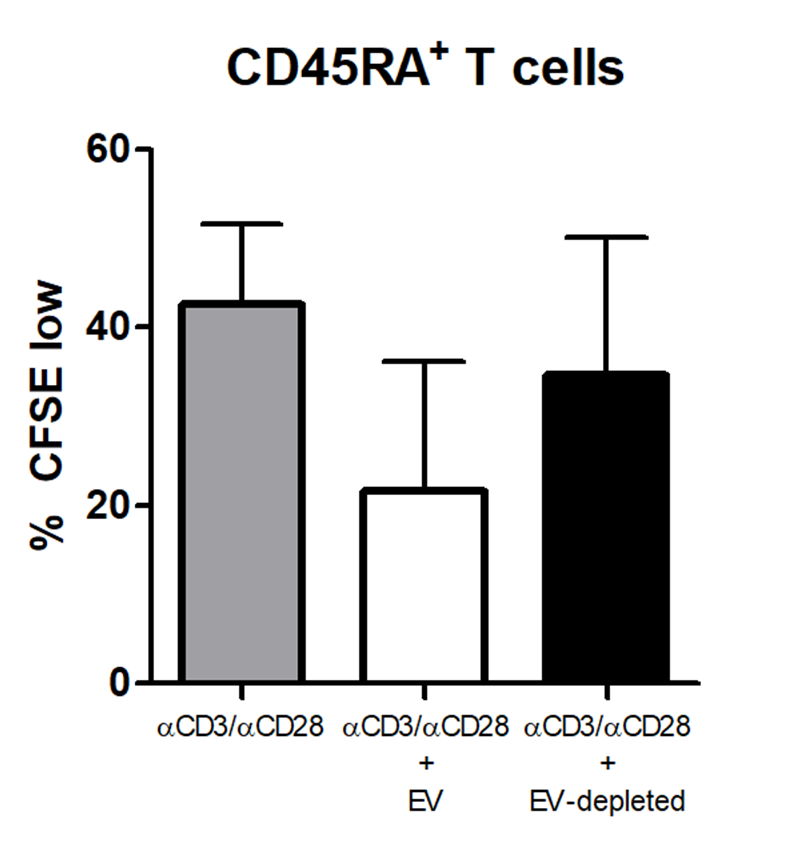


**Supplementary Fig. 11: Human milk EVs inhibit αCD3/αCD28 induced proliferation of CD4+CD45RA+ T cells.**

Purified CD45RA+CD45RO- CD4 T cells of 2 different donors were labeled with CFSE and stimulated with 1.5 µg/ml αCD3 and 1 µg/ml αCD28 in the presence or absence of milk EVs and EV-depleted controls for 6 days. Cells were subsequently harvested and CFSE dilution was determined by flow cytometry. Bars represent mean ± SD from the average of technical triplicates, each T cell donor was cultured with 3 different milk donors. Kruskal-Wallis and Dunn’s multiple comparison test revealed no significant differences.

­­­­­­­­­­­­­­­­­­­­­­­­_________________________________________________________________________________________________________________________________________________________________________________________________________________________________________________________________________________________________________________________


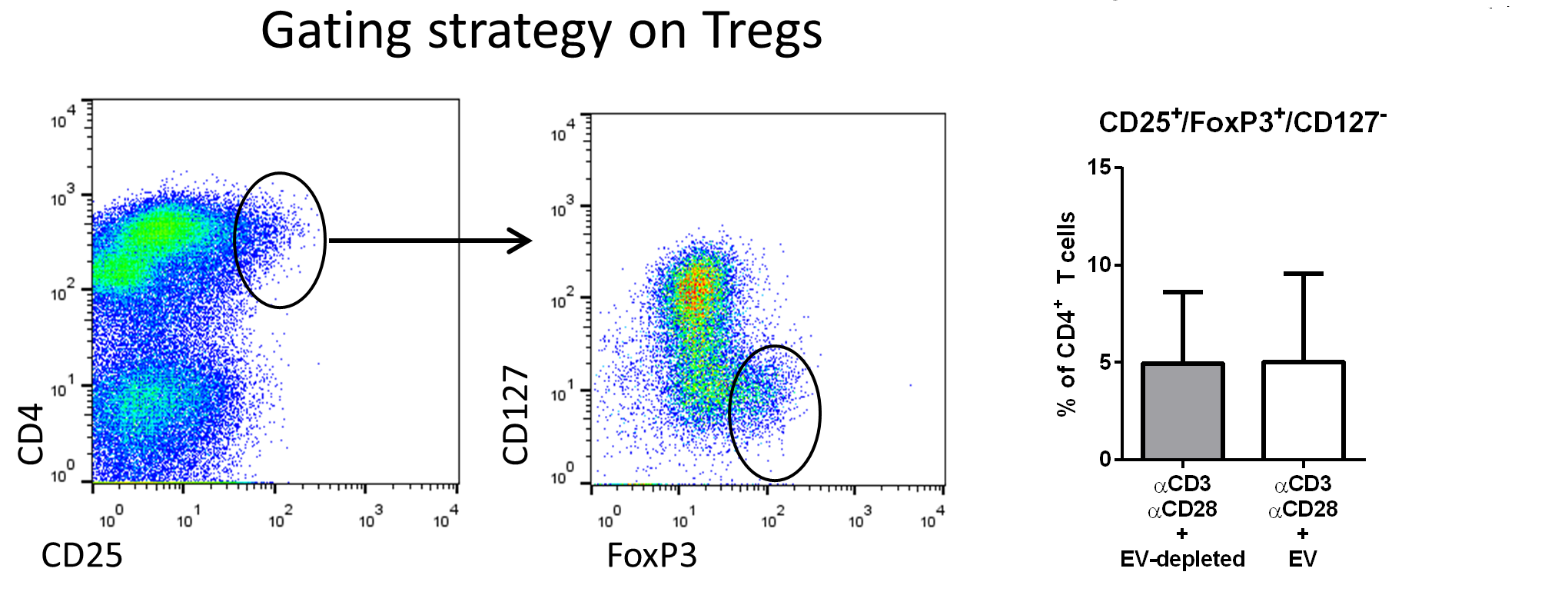


**Supplementary Fig. 12: Human milk EVs do not induce a regulatory T cell phenotype.**

Quantification of the typical CD4+CD25highFoxP3+CD127- phenotype of human regulatory T cells in PBMC following exposure to 1.5 µg/ml αCD3 in the presence of EV or donor-matched EV-depleted controls. Bars represent mean ± SD from a single technical triplicate of 4 independent experiments performed with 4 different PBMC donors and in total 6 different milk donors.

­­­­­­­­­­­­­­­­­­­­­­­­_________________________________________________________________________________________________________________________________________________________________________________________________________________________________________________________________________________________________________________________


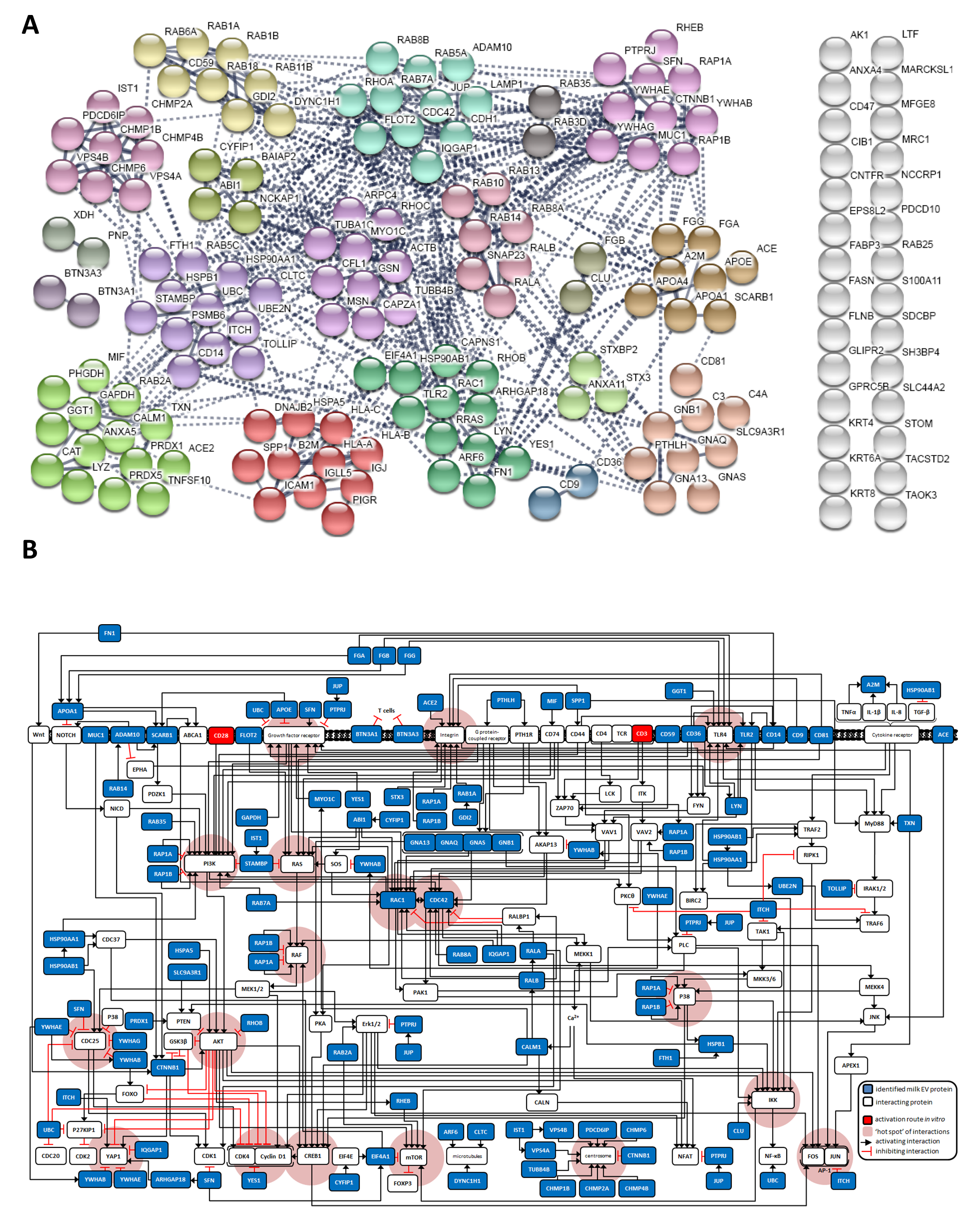


**Supplementary Fig. 13: Enrichment and protein-protein network analysis allowed complete integration of human milk EV proteins into signaling pathways associated to regulation of T cell activation and revealed interactions at multiple levels in the signaling cascades.**

A) Protein-protein interaction analysis on the identified milk EV proteins that linked to the selected GO-terms was performed (minimum required interaction score set to high confidence 0.700), followed by k-means clustering (showing 19 clusters) in order to visualize the most likely occurring construct clusters within the network. A total of 137 proteins formed protein-protein interactions (which is 83.0%) and 28 proteins had no interaction with any other milk EV protein (which is 17.0%). Only those proteins that were part of an interaction network were further investigated for validated links to T cell activation. B) Functional annotation analysis of previously identified milk EV proteins that link to the observed *in vitro* effects in Fig. 4: inhibition of T cell activation. The interaction of selected milk EV proteins (in blue) and cellular proteins (in white; either shown with their common gene name, or a synonym when widely used in literature) and the type of interaction (activating or inhibiting) within relevant signaling pathways are shown. If a protein has interactions with ≥ 6 other proteins, this node is shown in red as a ‘hotspot’. Although milk EV proteins were selected via relevant GO-terms, some proteins could not be linked to the specific signaling cascades and are not shown in B (see Supplementary File 3 for a full overview of the analysis).

­­­­­­­­­­­­­­­­­­­­­­­­_________________________________________________________________________________________________________________________________________________________________________________________________________________________________________________________________________________________________________________________


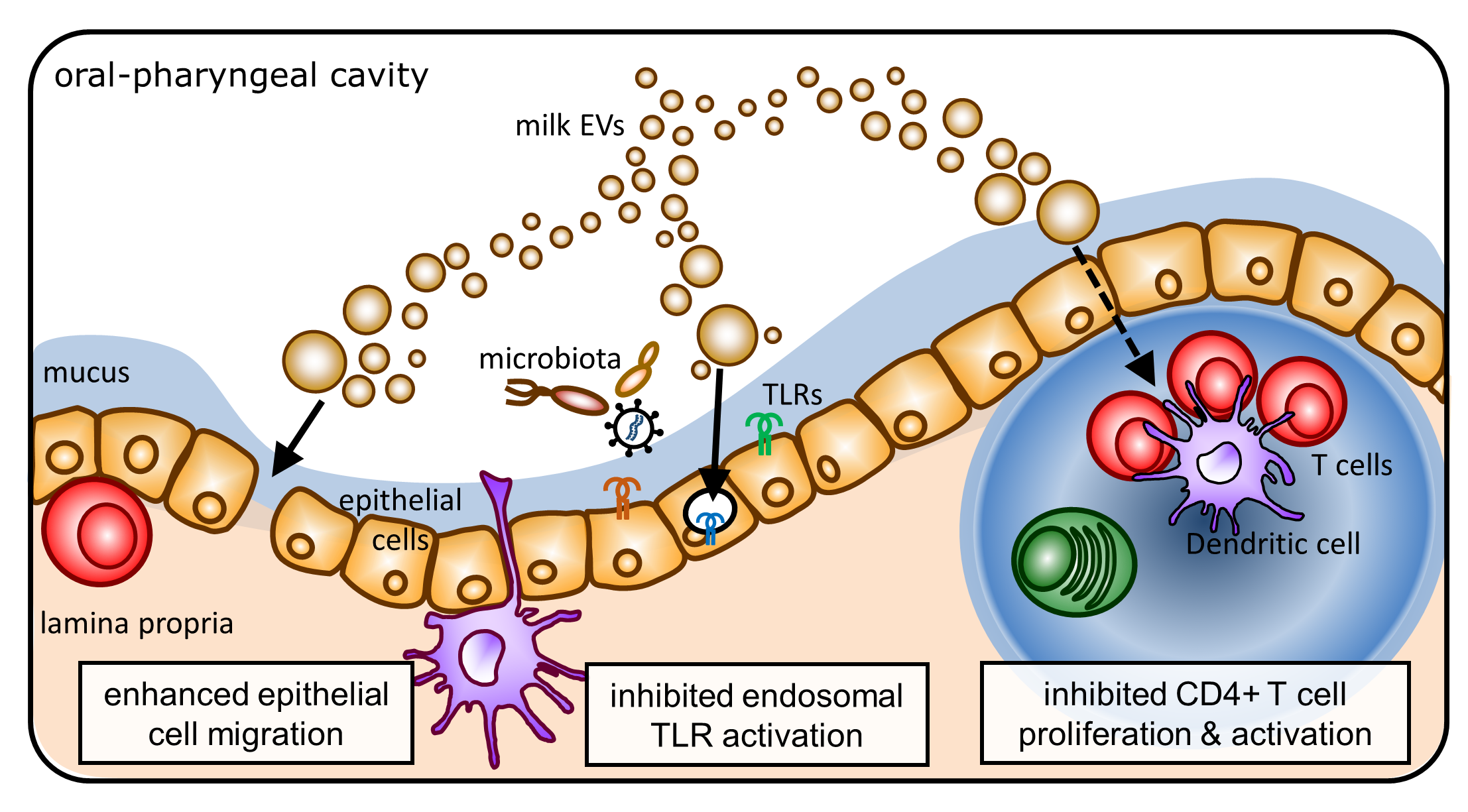


**Supplementary Fig. 14: Graphical conclusion.**

Milk extracellular vesicles (EVs) are a heterogeneous population of cell-derived multi-component nanoparticles that can interact and modulate a variety of cells present in the infant’s oral mucosa. Together with the microbiota, possible pathogens, food- and environmental components, maternal milk EVs interconnectedly communicate with these mucosal cells. The gingival epithelial cells must form a tight barrier in order to prevent breaches in the mucosal surface. Milk EVs enhance the migratory capacity of epithelial cells, allowing for rapid gap closure. Additionally, epithelial cells are part of the innate immune system as they scan for microbe-associated molecular patterns (MAMPs) via Toll Like Receptors (TLRs). Milk EVs specifically tune TLR signaling by inhibiting endosomal TLR activation. Since EVs can cross epithelial barriers, they can also reach adaptive immune cells which reside underneath the epithelial layer. In the presence of milk EVs, CD4+ helper T cells are inhibited in their activation but remain responsive to stimulation once milk EVs are absent. The collective cargo of the heterogeneous population of maternal milk EVs works in concert to target key hotspots of signaling networks in target cells to specifically fine-tune mucosal processes involved in barrier function and immunity, thereby creating a window for adaptation and regulated development.

­­­­­­­­­­­­­­­­­­­­­­­­_________________________________________________________________________________________________________________________________________________________________________________________________________________________________________________________________________________________________________________________
