# Supplementary material for: Human milk extracellular vesicles target nodes in interconnected signalling pathways that enhance oral epithelial barrier function and dampen immune responses
Source: J Extracell Vesicles. 2021 Mar 10;10(5):e12071. doi: 10.1002/jev2.12071 (PMC7944547; doi:10.1002/jev2.12071)
Supplement: Supplementary file 2 — Supplementary information [file JEV2-10-e12071-s002.pptx]

## Slide 1
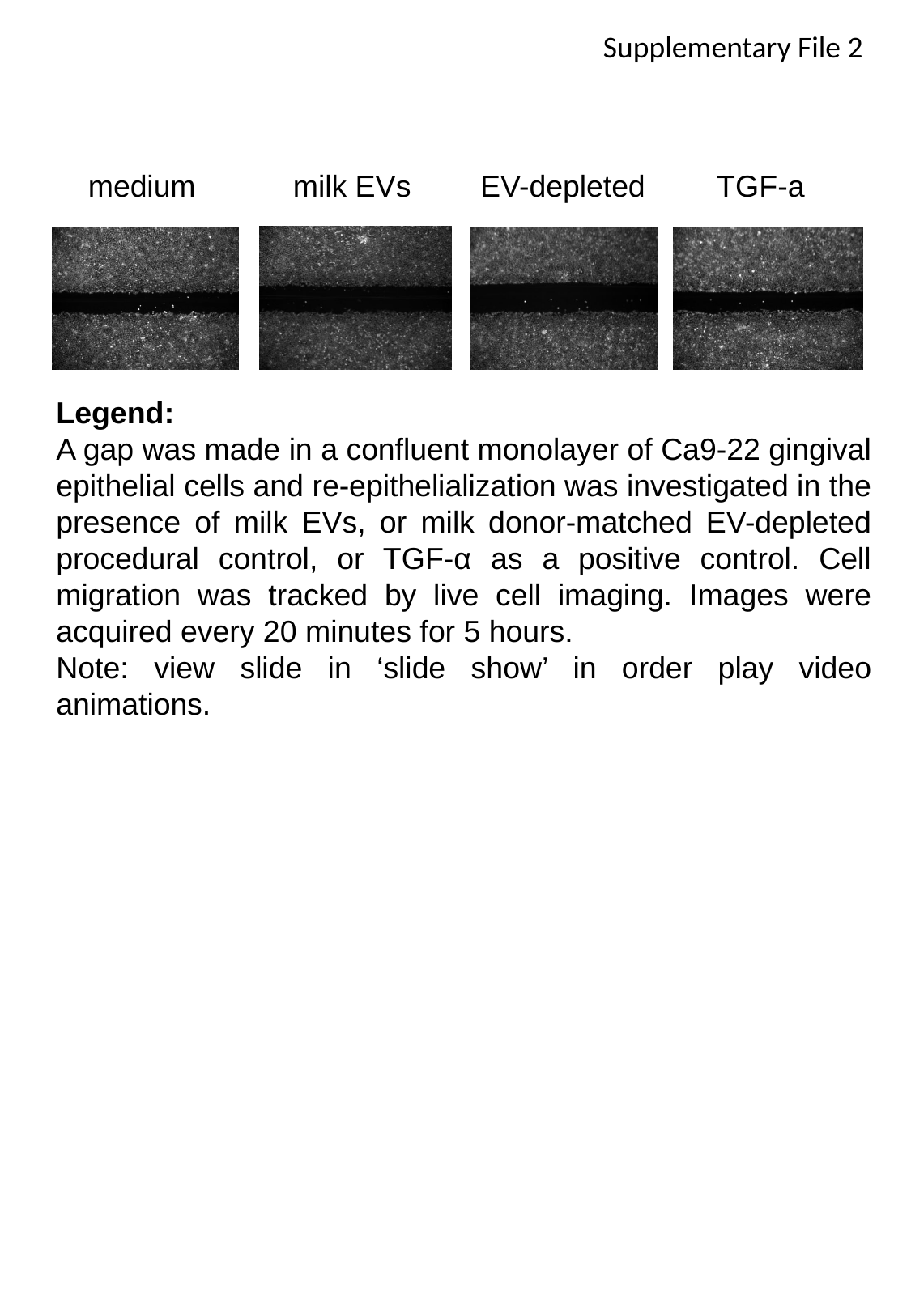

Supplementary File 2
medium
EV-depleted
milk EVs
TGF-a
Legend:
A gap was made in a confluent monolayer of Ca9-22 gingival epithelial cells and re-epithelialization was investigated in the presence of milk EVs, or milk donor-matched EV-depleted procedural control, or TGF-α as a positive control. Cell migration was tracked by live cell imaging. Images were acquired every 20 minutes for 5 hours.
Note: view slide in ‘slide show’ in order play video animations.
